# PJA2 Suppresses Colorectal Cancer Progression by Controlling HDAC2 Degradation and Stability

**DOI:** 10.1002/advs.202401964

**Published:** 2025-02-10

**Authors:** Zhihao Chen, Chaofan Peng, Chi Jin, Ye Wang, Tuo Wang, Peng Yang, Wen Peng, Qingyang Sun, Hengjie Xu, Hongxu Nie, Xiaowei Wang, Junwei Tang, Yueming Sun, Yifei Feng

**Affiliations:** ^1^ Department of General Surgery Colorectal Institute of Nanjing Medical University The First Affiliated Hospital of Nanjing Medical University Nanjing 210029 P. R. China; ^2^ Jiangsu Province Engineering Research Center of Colorectal Cancer Precision Medicine and Translational Medicine Nanjing P. R. China; ^3^ The First School of Clinical Medicine Nanjing Medical University Nanjing P. R. China

**Keywords:** colorectal cancer, HDAC2, IFIT2, PJA2, ubiquitination

## Abstract

PJA2 is documented to degrade various substrates. Nevertheless, the role of PJA2 as an E3 ubiquitin‐protein ligase in colorectal cancer (CRC) progression remains unexplored. The correlation between PJA2 mRNA levels and clinical characteristics is investigated using data from The Cancer Genome Atlas (TCGA) database. Quantitative real‐time polymerase chain reaction (qRT‐PCR) and immunohistochemistry (IHC) are utilized to evaluate PJA2 expression levels in CRC tissues. The biological functions of PJA2 are confirmed through colony formation assays and azoxymethane/dextran sulfate sodium (AOM/DSS) mouse model of CRC, among other experimental approaches. The underlying molecular mechanisms of PJA2 action are elucidated using RNA sequencing (RNA‐seq), co‐immunoprecipitation (co‐IP), proximity ligation assay (PLA), and chromatin immunoprecipitation (ChIP). Our research discovered that PJA2 is downregulated in CRC tissues and decreased PJA2 expression correlates with poor prognosis. Functionally, in vivo and in vitro experiments uncovered that PJA2 inhibits tumor cell proliferation and promotes apoptosis. Mechanistically, PJA2 recognized histone deacetylase 2 (HDAC2) via its RING‐B‐box domain (RBD) and bind to the N‐terminal of HDAC2, facilitating ubiquitination at the lysine 90 (K90) residue. PJA2‐mediated degradation of HDAC2 counteracts the transcriptional repression of the interferon‐induced protein with the tetratricopeptide repeats (IFIT) family, thereby suppressing CRC progression. The data demonstrates that PJA2 suppresses CRC progression through the PJA2/HDAC2/IFIT axis, and its expression is regulated by HDAC2, thus constituting a positive feedback loop. Consequently, PJA2 may serve as a potential therapeutic target for CRC, and interrupting this feedback loop can represent a viable treatment strategy to restrain CRC progression.

## Graphical abstract

PJA2, an E3 ubiquitin‐protein ligase, actively restrains colorectal cancer (CRC) progression. It targets histone deacetylase 2 (HDAC2) for degradation, which in turn activates the interferon‐induced protein with tetratricopeptide repeats (IFIT) family, effectively hindering tumor growth and promoting apoptosis. Notably, reduced PJA2 expression correlates with poor prognosis, highlighting its potential as a therapeutic target for CRC.

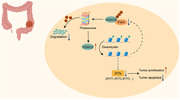

## 1 Introduction

CRC ranks as the third most prevalent malignancy and the third leading cause of cancer‐related deaths in both males and females, according to the latest statistics.^[^ ^1^ ^]^ Despite advancements in surgical interventions and chemotherapy, the persistently high global mortality and morbidity rates continue to burden millions of CRC patients. Therefore, there is an urgent need to elucidate the underlying mechanisms and identify novel therapeutic targets in CRC, which could significantly enhance the prognosis for these patients.

Ubiquitination, an extensively occurring post‐translational modification (PTM) in colorectal cancer, spatially and temporally regulates the expression of various substrate proteins and plays a crucial role in the development and distant metastasis of colorectal cancer.^[^ ^2^, ^3^ ^]^ Within the ubiquitin‐proteasome system (UPS), E1, E2, and E3 enzymes sequentially transfer ubiquitin to a substrate. Among these, E3 ubiquitin‐protein ligases are critical in recognizing and determining which substrates to degrade. The RING finger protein family, characterized by the presence of the N‐terminal RING domain, is the largest and most significant E3 ubiquitin ligase family, comprising >340 validated members.^[^ ^4^ ^]^ Praja Ring Finger Ubiquitin Ligase 2 (PJA2), a member of the mammalian RING finger protein family, is broadly expressed in diverse organs and tissues, including the colorectum. Previous reports have shown that PJA2 accelerates the ubiquitin‐related proteolysis of inhibitory PKA regulatory subunits, thereby releasing and activating the catalytic subunits and subsequent downstream pathways.^[^ ^5^ ^]^ Additionally, PJA2 increases the ubiquitination of other functional proteins that control the tumor signaling network, metabolic reprogramming, and neuronal signal transduction.^[^ ^6^, ^7^, ^8^ ^]^ However, the molecular mechanism of PJA2 in colorectal cancer progression remains unexplored. Understanding this mechanism could not only provide a new molecular marker for the diagnosis of colorectal cancer but also improve the clinical efficacy of corresponding targeted therapeutic drugs.

Deacetylation of histones plays an indispensable role in epigenetic regulation, and emerging evidence indicates that this process is widely involved in various biological processes.^[^ ^9^, ^10^ ^]^ As the core component of the corresponding enzymes, histone deacetylases (HDACs) catalyze the removal of acetyl groups from lysine‐rich histone residues, mediating transcriptional inhibition. HDACs are divided into different subgroups, and HDAC2 belongs to the classical deacetylases. HDAC2 has been shown to be an independent prognostic factor associated with poor outcomes in various cancers, including oral, prostate, and gastric cancer.^[^ ^11^, ^12^, ^13^ ^]^ Zhu et al. found increased HDAC2 expression in most human colon cancer tissues and the intestinal mucosa and polyps of APC‐deficient mice, and they demonstrated that HDAC2 is required to prevent apoptosis in colon cancer cells.^[^ ^14^ ^]^ However, the specific molecular mechanism by which HDAC2 regulates apoptosis and its ubiquitination modification in colorectal cancer remains unclear. Investigating the PJA2‐HDAC2 axis is of significant importance for the potential clinical application of the HDAC inhibitor Indapamide.

Here, we demonstrate for the first time that PJA2 is downregulated in CRC and that patients with low PJA2 expression tend to have a poorer prognosis. Both in vitro and in vivo experiments confirm that PJA2 suppresses tumor proliferation and promotes tumor apoptosis. We validate that downregulated PJA2 diminishes the ubiquitin‐related degradation of HDAC2 and encourages the transcriptional activation of the IFIT family. Crucially, the increase in HDAC2 due to reduced poly‐ubiquitination and degradation strengthens the transcriptional repression of PJA2, generating a feedback loop that exacerbates CRC progression. Understanding the role of PJA2 in CRC progression provides vital insight into CRC pathogenesis and should be considered in the development of therapeutic interventions.

## 2 Results

### 2.1 A Low Level of PJA2 is Associated with CRC Progression

To screen and identify ubiquitin ligases that determine CRC progression, we analyzed the expression matrices of CRC and adjacent normal tissues in public datasets with large sample sizes (*n*>190) and high gene coverage. Through differential expression gene analysis, we identified 2 conservatively upregulated and 3 downregulated differentially expressed ubiquitin ligase genes in all 5 selected datasets. Notably, only PJA2 and TRIM36 were significantly associated with survival prognosis based on the TCGA database (Figure S1A, Supporting Information). Using qRT‐PCR, we detected the expression levels of PJA2 and TRIM36 mRNA in 20 pairs of colorectal cancer tissue samples from our center and found that the expression of PJA2 in colorectal cancer tissues was more significantly different (Figure S1B,C, Supporting Information). Therefore, we focused on the role of the ubiquitin ligase PJA2 in the development of colorectal cancer.

**Figure 1:**
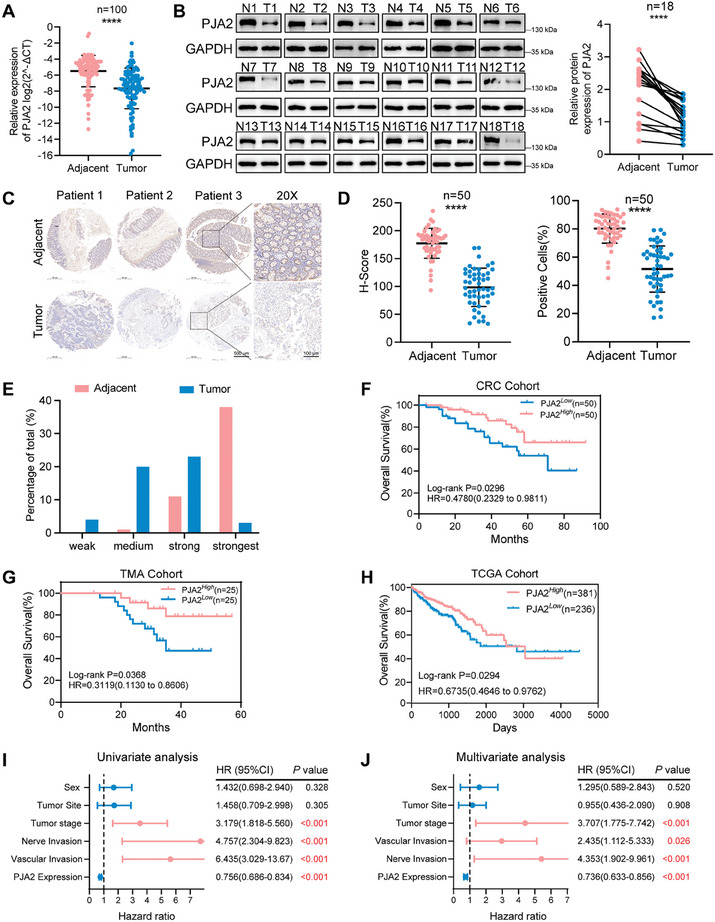
PJA2 is downregulated in CRC and associated with poor prognosis. A) The expression at mRNA levels of PJA2 was detected by qRT‐PCR in 100 paired CRC tissues and adjacent tissues (CRC cohort), with GAPDH as the internal reference gene. B) The expression at protein levels of PJA2 was detected by WB in 18 paired CRC tissues and adjacent tissues, with GAPDH as the internal reference gene. C–E) The protein level of PJA2 detected by IHC in the TMA of 50 CRC patients’ samples (TMA cohort). C) representative images of PJA2 expression, D) the relative statistical analysis of the H‐Score (left) and the proportion of PJA2 immunohistochemically positive cells (right), E) the distribution of IHC staining detected by IHC scores. F–H0 The survival analysis of PJA2 expression. The Kaplan‐Meier Overall Survival analysis of PJA2 expression in the CRC cohort (F), TMA cohort G), and TCGA cohort H). I) Univariate survival analysis of PJA2 in the CRC cohort. J) Multivariate survival analysis of PJA2 in the CRC cohort. All data are shown as the means ± SD of three independent experiments and a *p*‐value under 0.05 was considered to be sReferencestatistically significant. ns *p* > 0.05, **p < 0.01, ***p < 0.001, **References**p<0.0001.

To better identify the expression level of PJA2 in CRC, we detected the mRNA and protein levels of PJA2 in samples from colorectal cancer patients: 100 samples for qPCR (CRC cohort), 18 cases for WB, and 50 samples for IHC (TMA cohort). We found that both mRNA and protein levels of PJA2 were downregulated in tumor tissues (**Figure** **1**A,B). Similar results were observed when comparing tissue microarrays (TMA) consisting of 50 paired CRC samples (Figure 1C), as evidenced by lower H‐scores and proportions of positive cells in tumor tissues (Figure 1D), as well as the higher degrees of immunohistochemistry (IHC) staining intensity (Figure 1E). Additionally, the association between the expression of PJA2 and the clinicopathological characteristics of the CRC cohort indicated that PJA2 expression was negatively correlated with pT stage, lymph node metastasis, vascular invasion, and nerve invasion, suggesting that PJA2 may be a crucial target in suppressing CRC progression (Table S1, Supporting Information). Furthermore, Kaplan‐Meier survival analyses of the CRC and TMA cohorts uncovered that patients with high expression of PJA2 had better overall survival (Figure 1F,G). Similarly, we also found that patients with low PJA2 expression tended to exhibit worse overall survival in the TCGA dataset (Figure 1H). Cox univariate and multivariate analyses further illustrated that the expression of PJA2 is an independent prognostic factor for CRC patients (Figure 1I,J). Overall, these results confirm that PJA2 is downregulated in CRC and suggest that it could be a potential diagnostic biomarker for CRC.

### 2.2 PJA2 Suppresses Proliferation and Promotes Apoptosis of CRC Cells

Given that tumor proliferation is the most classical feature of the malignant phenotype, the role of PJA2 in CRC proliferation was explored. Detection results showed that SW480 and RKO cell lines had high expression of PJA2, while HCT116 and DLD‐1 cell lines had low expression (Figure S1D, Supporting Information). After constructing the plasmids, the above cells were infected using a lentivirus‐mediated approach. Results showed that Sh‐PJA2#1 and Sh‐PJA2#3 had significant knockdown efficiency in SW480 and RKO cells (Figure S1E, Supporting Information), and the expression of PJA2 was stably overexpressed in HCT116 and DLD‐1 cells (Figure S1F, Supporting Information).

To assess the function of PJA2 in regulating cell proliferation, in vitro assays were conducted. The consumption of PJA2 in SW480 and RKO accelerated proliferation curves, increased colony formation, and elevated the proportion of EDU‐positive cells (Figure S2A,C,E, Supporting Information). Conversely, the overexpression of PJA2 in HCT116 and DLD‐1 suppressed tumor cell proliferation (Figure S2B,D,F, Supporting Information). Besides, flow cytometric assays revealed that downregulated PJA2 decreased the ratio of apoptotic cells in SW480 and RKO cells (Figure S2G, Supporting Information). Conversely, the overexpression of PJA2 caused an increased rate of apoptosis in HCT116 and DLD‐1 cells (Figure S2H, Supporting Information). Collectively, these results suggest that PJA2 functions as a tumor suppressor by inhibiting proliferation and promoting apoptosis in CRC.

**Figure 2:**
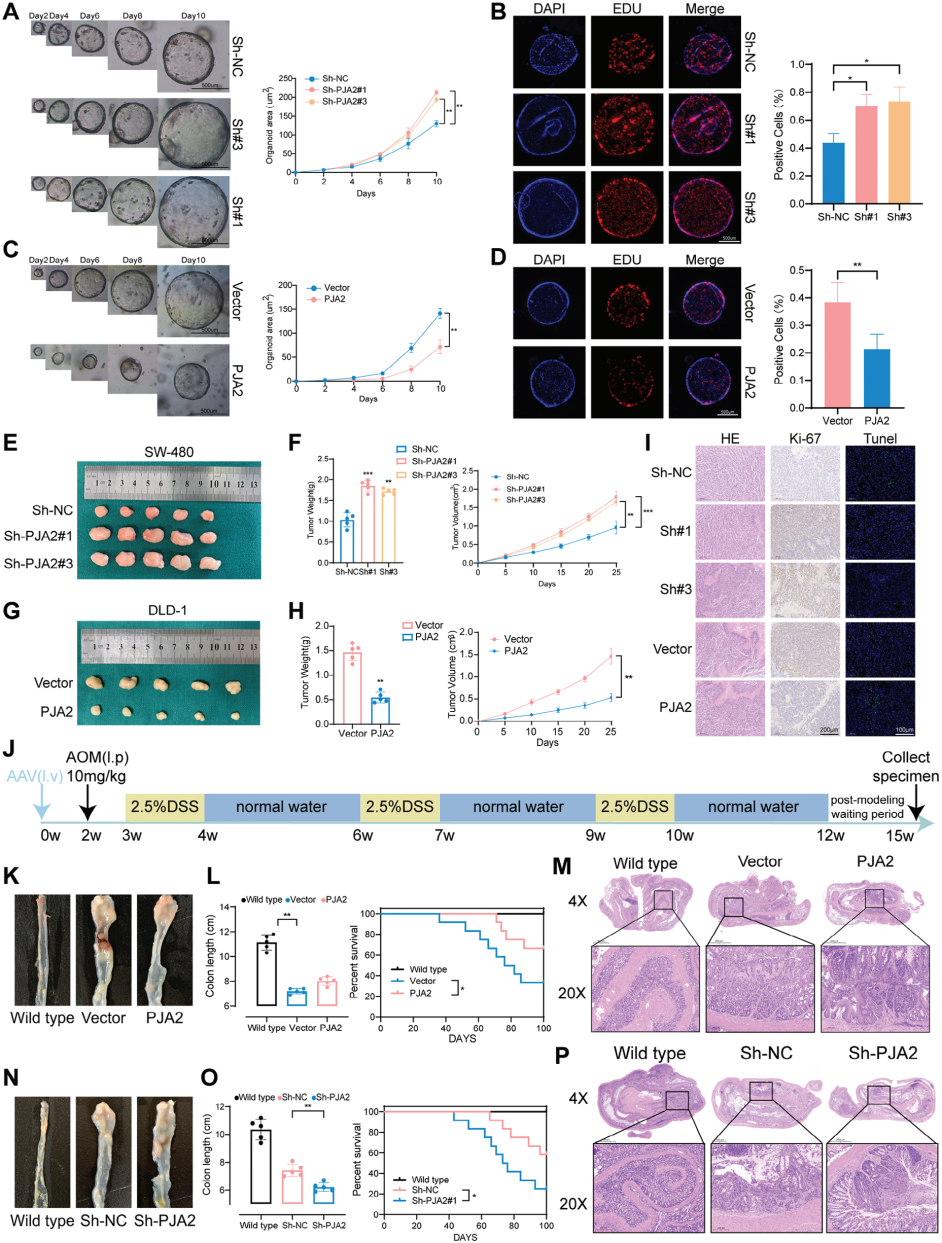
PJA2 suppresses proliferation and promotes apoptosis of CRC cells in vitro *and* in vivo. A) Representative images (left) and growth curve (right) of CRC PDO models. The PDOs were detected with or without the knockdown of PJA2 expression for 10 days (*n*  =  3), scale bar: 500 µm. B) EdU staining assays were conducted to evaluate the proliferation ability of organoids with or without the knockdown of PJA2. C) Representative images (left) and growth curve (right) of CRC PDO models. The PDOs were detected with or without the overexpression of PJA2 expression for 10 days (*n*  =  3), scale bar: 500 µm. D) EdU staining assays were conducted to evaluate the proliferation ability of organoids with or without the overexpression of PJA2. E) Representative photographs of subcutaneous xenograft tumors were obtained from nude mice with or without the knockdown of PJA2. (*n* = 5 for each group). F) Statistical plots of tumor weight (left) and tumor volume (right) analysis. The tumor volumes were measured every five days, and the tumor weights were analyzed after the mice were sacrificed. G) Representative photographs of subcutaneous xenograft tumors were obtained from nude mice with or without the overexpression of PJA2. (*n* = 5 for each group). H) Statistical plots of tumor weight (left) and tumor volume (right) analysis. The tumor volumes were measured every five days, and the tumor weights were analyzed after the mice were sacrificed. I) Representative photographs of H&E, Ki‐67, and Tunel staining in xenograft tumors. J) Schematic diagram of AOM/DSS‐induced colorectal cancer in C57/B6 mice. K) Representative images of colons in different groups (n = 5 for each group): wild type, AAV‐vector + DSS, AAV‐PJA2 + DSS. L) The colon lengths analysis of colons (left) and the Kaplan–Meier survival analysis (right) in indicated groups. M) H&E staining of colons in indicated groups. N) Representative images of colons in different groups (*n* = 5 for each group): wild type, AAV‐Sh‐NC + DSS, AAV‐Sh‐PJA2 + DSS. O. The colon lengths analysis of colons (left) and the Kaplan–Meier survival analysis (right) in indicated groups. P) H&E staining of colons in indicated groups. All data are shown as the means ± SD of three independent experiments and a *p*‐value under 0.05 was considered to be statistically significant. ns *p* > 0.05, **p < 0.01, ***p < 0.001, ****p<0.0001.

To better simulate the biological phenotype of PJA2 in human colorectal cancer, we extracted patient‐derived organoids (PDOs) from colorectal cancer tissue. By comparing hematoxylin and eosin (H&E) staining and immunohistochemistry for Ki‐67 and carcinoembryonic antigen (CEA) between human colorectal cancer tissue and PDOs, we confirmed the reliable origin of the PDOs (Figure S3A, Supporting Information). Additionally, we observed the transfection efficiency of PJA2 knockdown and overexpression lentiviruses tagged with GFP using fluorescence microscopy (Figure S3B, Supporting Information). By comparing the growth curves and the ratio of EDU‐positive cells in the organoids, we found that the growth and proliferation of PDOs were significantly stimulated after PJA2 knockdown (**Figure** **2**A,B). Conversely, the organoids in the overexpression group displayed weaker growth curves and reduced proliferative activity (Figure 2C,D).

To validate the effect of PJA2 on colorectal cancer cell proliferation in vivo, lentivirus‐transfected cells were subcutaneously injected into nude mice. Results showed that cells with depleted PJA2 promoted tumor proliferation, as evidenced by greater tumor weight and volume (Figure 2E,F). Conversely, cells with PJA2 overexpression exhibited opposite effects (Figure 2G,H). Furthermore, the PJA2 knockdown group presented elevated Ki‐67 staining and decreased Tunel staining, while the PJA2 overexpression group exhibited decreased Ki‐67 staining and elevated Tunel staining (Figure 2I).

To better verify the role of PJA2 in CRC tumorigenesis, AOM/DSS‐induced colon cancer (CAC) models were constructed in C57/BL6 mice (Figure 2J). Adeno‐associated virus (AAV) was injected two weeks before CAC induction to alter the expression of PJA2, with the transduction efficiency validated at the protein level (Figure S3C, Supporting Information). Elevation of PJA2 significantly suppressed the progression of CAC models (Figure 2K; Figure S3D, Supporting Information), as evidenced by less colon shortening, more weight gain, and smaller cancerous nodes (Figure 2I; Figure S3E,F, Supporting Information). Besides, the H&E staining results also indicated that PJA2 could restrain the proliferation and progression of colorectal cancer (Figure 2M). Conversely, the Sh‐PJA2 group promoted CRC proliferation and showed opposite trends in these observational indices (Figure 2N–P; Figure S3G–I, Supporting Information). Consequently, upon expanding the sample size for the model establishment, the results demonstrated that mice overexpressing PJA2 exhibited significantly improved survival rates, whereas those with PJA2 knockdown displayed higher mortality rates (Figure 2I,O). Taken together, both in vivo and in vitro experiments confirmed that PJA2 acts as an inhibitor of tumor progression by suppressing proliferation and promoting apoptosis.

### 2.3 PJA2 Increases the Transcriptional Activity of the IFIT Family

**Figure 3:**
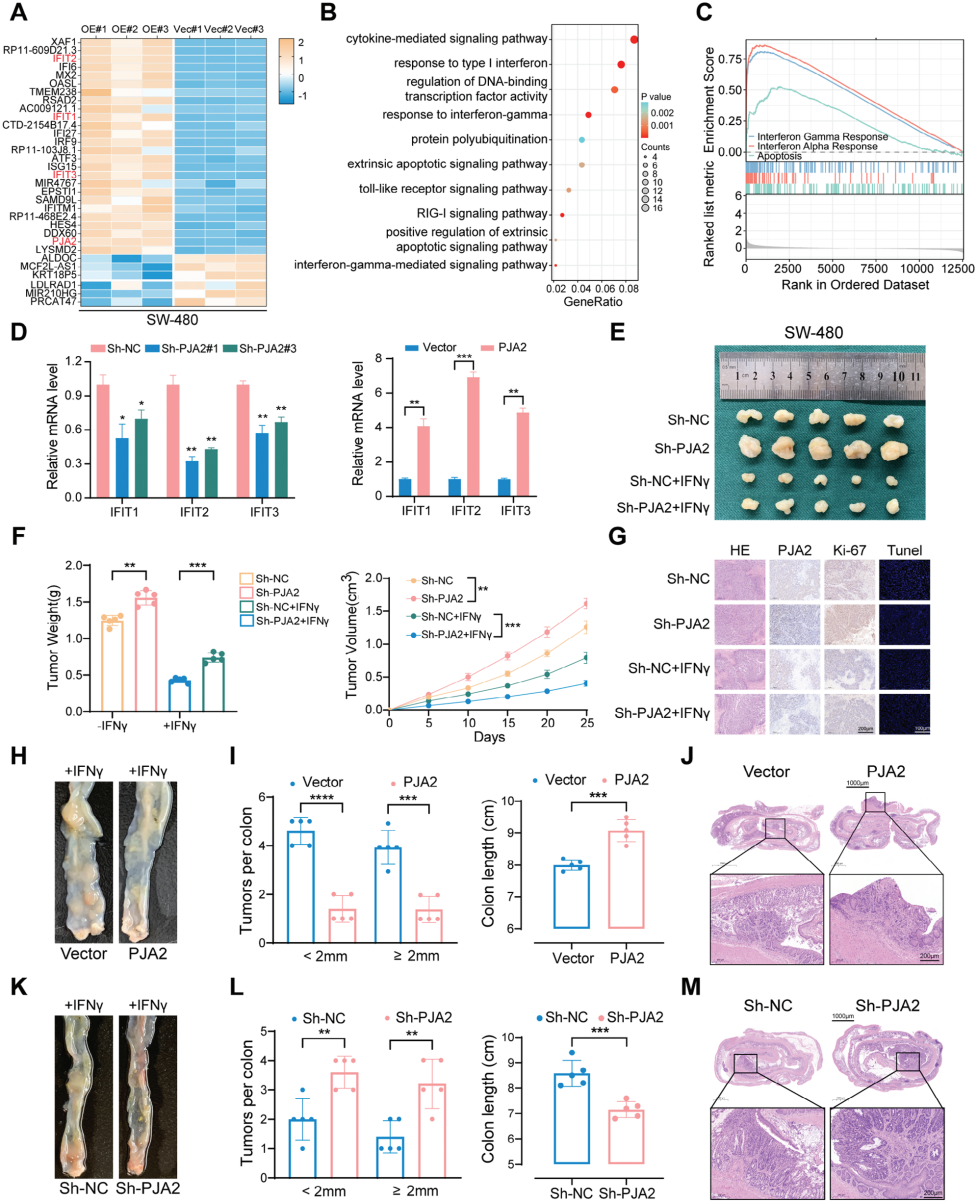
PJA2 increases the transcriptional activity of IFITs. A) Heatmap representing the most significantly regulated genes (fold change>2 or <2, *p*<0.05) detected in RNA‐seq analysis with overexpression of PJA2 in SW480 cells. B) GO enrichment analysis of 320 significantly regulated genes identified by RNA‐seq. C) GSEA analysis of 15440 altered genes identified by RNA‐seq. D) The mRNA levels of the IFIT family in SW480 cells with PJA2 knockdown (left) and PJA2 overexpression (right) were detected by qPCR. E) Representative images of subcutaneous xenograft tumors (*n* = 5 for each group) were obtained from nude mice after the knockdown of PJA2 and injection of interferon. F) Statistical plots of tumor weight (left) and tumor volume (right) analysis. The tumor volumes were measured every five days, and the tumor weights were analyzed after the mice were sacrificed. G) Representative photographs of H&E, IHC, and Tunel staining in xenograft tumors. The protein levels of Ki67 and PJA2 in xenograft tumors were detected by IHC. H. Representative images of colons in different groups (*n* = 5 for each group): wild type, AAV‐vector + DSS + mouse interferon injection, AAV‐PJA2 + DSS+ mouse interferon injection. I) The tumor number analysis (left) and the colon lengths analysis (right) of indicated groups. J) H&E staining of colons in indicated groups. K) Representative images of colons in different groups (*n* = 5 for each group): wild type, AAV‐Sh‐NC + DSS + mouse interferon injection, AAV‐Sh‐PJA2 + DSS+ mouse interferon injection. L) The tumor number analysis (left) and the colon lengths analysis (right) of indicated groups. M) H&E staining of colons in indicated groups. All data are shown as the means ± SD of three independent experiments and a *p*‐value under 0.05 was considered to be statistically significant. ns *p* > 0.05, **p < 0.01, ***p < 0.001, ****p<0.0001.

To explore the mechanism by which PJA2 regulates the progression of CRC, RNA‐seq was performed to compare differences between PJA2‐overexpressing cells and vector control cells in SW480. After analyzing significantly differentially expressed genes (fold change > 2 or < −2, *p* < 0.05), 78 genes were identified, and the top 40 differentially expressed genes were displayed (**Figure** **3**A). Among these genes, the expression of the IFIT family was remarkably upregulated in the OE‐PJA2 group, which was also verified by the positive correlation between mRNA levels of PJA2 and the IFIT family in colorectal adenocarcinoma at the Chipbase database (Figure S4A,C,E, Supporting Information). In probing the biological function of PJA2, Gene ontology (GO) analysis of 320 significantly dysregulated genes showed that enriched biological processes included cytokine‐mediated signaling pathway, protein polyubiquitination, extrinsic apoptotic signaling pathway, and response to interferon (Figure 3B). Additionally, Gene set enrichment analysis (GSEA) of 15440 altered genes in the OE‐PJA2 group indicated that PJA2 participates in the process of interferon‐gamma response and apoptosis (Figure 3C), consistent with the results of GSEA analysis in the TCGA CRC dataset (Figure S4G,H, Supporting Information). Moreover, the depletion of PJA2 decreased the transcriptional activity of the IFIT family and the overexpression of PJA2 exhibited opposite phenomena, particularly in the regulation of IFIT2 (Figure 3D).

As previously reported, the IFIT family (IFN‐induced protein with tetratricopeptide repeats) was primarily found in antiviral immune responses. An expanding body of evidence demonstrates that the IFIT family is broadly implicated in cellular biology, encompassing processes such as apoptosis, proliferation, and tumorigenesis.^[^ ^15^, ^16^, ^17^ ^]^ Within the realm of oncology, IFN γ has increasingly been reported as a critical driver of the tumor‐suppressive effects mediated by IFIT family proteins.^[^ ^18^, ^19^ ^]^ Further in vitro phenotypic experiments showed that the knockdown of PJA2 could restore the anti‐proliferation and pro‐apoptotic effect of interferon on SW480 and RKO cells (Figure S5A,C,E, Supporting Information). Conversely, the overexpression of PJA2 could enhance the anti‐proliferation and pro‐apoptotic function of interferon on DLD‐1 and HCT116 cells (Figure 3E; Figure S5B,D,F, Supporting Information).

Compared to the control group, the knockdown of PJA2 restrained the tumor suppression effect of interferon, as evidenced by larger tumor weight and volume, elevated Ki‐67 staining, and decreased Tunel staining (Figure 3E–G). Consistently, the overexpression of PJA2 amplified the tumor suppression function of interferon (Figure S6A–D, Supporting Information). During the process of the AOM/DSS model, interferon was intraperitoneally injected once a week (Figure 2J). With the overexpression of PJA2, the inhibition of tumor growth caused by interferon could be amplified (Figure 3H–J; Figure S6E, Supporting Information), and the depletion of PJA2 caused contrary phenomena (Figure 3K–M; Figure S6F, Supporting Information). Collectively, these data indicated that PJA2 promotes the transcription of the IFIT family and amplifies the anti‐tumor effect of interferon.

### 2.4 HDAC2 is Identified as a New Substrate of PJA2

**Figure 4:**
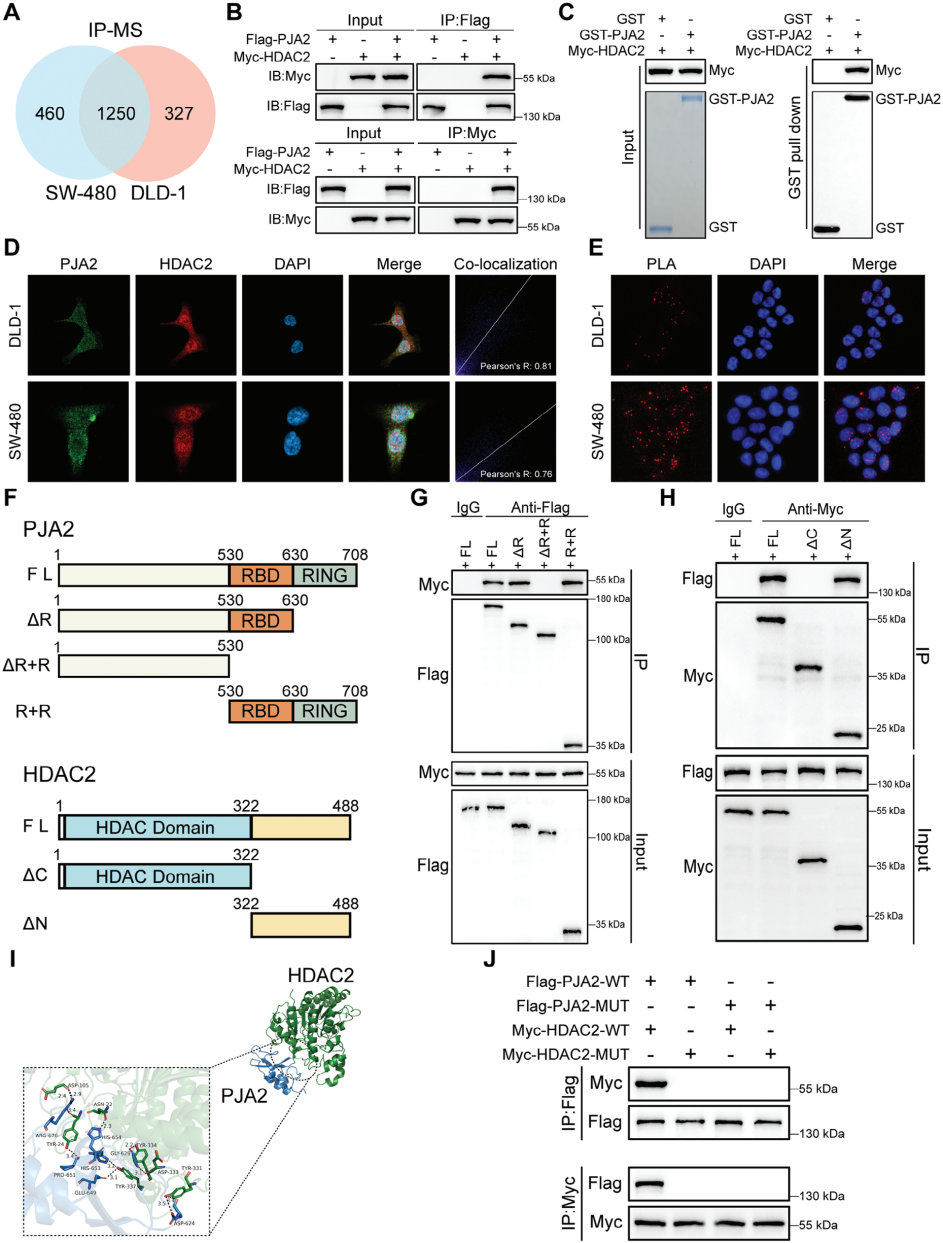
PJA2 physically interacts with HDAC2. A) The results of immunoprecipitation‐mass spectrometry (IP‐MS) analysis in SW480 and DLD‐1 cells with PJA2 overexpression. B) HEK293T cells transfected with indicated plasmids for 24 h were treated with MG132 (10 µM) for 8 h. The exogenous interaction between PJA2 and HDAC2 was detected by Co‐IP and western blotting assays. C) Western blot analysis of HDAC2 binding to purified GST and GST‐tagged PJA2 using Myc antibody (top). GST and GST‐tagged PJA2 were visualized by staining with Coomassie brilliant blue R‐250 (bottom). D) PJA2 and HDAC2 were detected by IF staining in SW480 and DLD‐1 cells (left). The co‐localization analysis was performed by ImageJ (right). E) Proximity ligation assays (PLA) were performed using anti‐PRMT6 and anti‐p62 antibodies in DLD‐1 and SW480 cells. Red signals represent PLA signals, indicating the proximity of the targeted proteins, while blue signals denote the cell nuclei. F) Schematic representation of PJA2 and HDAC2 truncations. G) IP and western blot analysis of the interaction between FLAG‐tagged truncated PJA2 and Myc‐tagged HDAC2 proteins in HEK293T cells. Cell extracts were IP with an anti‐Flag Ab. H) IP and western blot analysis of the interaction between FLAG‐tagged PJA2 and Myc‐tagged truncated HDAC2 proteins in HEK293T cells. Cell extracts were IP with an anti‐Myc Ab. I) The structure of PJA2 and HDAC2 proteins was visualized by SWISS‐MODEL, and the analysis of the physical interaction was performed by HDOCK. J) IP and western blot analyses show the interactions between FLAG‐tagged mutated PJA2 and Myc‐tagged mutated HDAC2 in HEK293T cells. The mutations were designed according to the scheme presented in Figure S7H (Supporting Information). All data are shown as the means ± SD of three independent experiments and a *p*‐value under 0.05 was considered to be statistically significant. ns *p* > 0.05, **p < 0.01, ***p < 0.001, ****p<0.0001.

To seek the specific substrate of PJA2 responsible for its ubiquitin‐protein ligases function, SW480 and DLD‐1 with PJA2 overexpressed were selected to make immunoprecipitation‐mass spectrometry (IP‐MS) analysis, and 1250 proteins were identified as potential PJA2‐interacting proteins (**Figure** **4**A). We listed the top ten intersections, and after excluding nonspecific binding confounders, HDAC2 was selected as the target protein of PJA2 (Figure S7A,B, Supporting Information). This selection was based on the high abundance of HDAC2 and previous studies reporting that HDAC2 regulates the transcriptional activity of SMAD3 to maintain the tumorigenic potential of brain tumor stem cells (BTSCs).^[^ ^20^ ^]^ Co‐IP experiments verified that endogenously expressed PJA2 and HDAC2 could interact with each other in SW480 and DLD‐1 cells (Figure S7C,D, Supporting Information). Similarly, flag‐tagged PJA2 and myc‐tagged HDAC2 were exogenously overexpressed in HEK‐293T cells, and their interaction was also observed (Figure 4B). GST pull‐down assays also confirmed that PJA2 physically interacts with HDAC2 (Figure 4C). Immunofluorescence (IF) assays indicated that PJA2 co‐localizes with HDAC2 in both the cytoplasm and nucleus, especially in the nucleus (Figure 4D). Furthermore, the results of proximity ligation assay (PLA) assays also verified that the signals indicating the proximity of PJA2 and HDAC2 were mainly concentrated in the nucleus, and the signals were stronger in the SW480 cell line, which had a high expression of PJA2 (Figure 4E). Based on their structures, the full‐length PJA2 protein was divided into three fragments, and the HDAC2 protein was cut into two segments (Figure 4F). Co‐IP experiments disclosed that the RBD domain (residues 530–630) was the primary fragment of PJA2 contributing to the combination with HDAC2 (Figure 4G), and the PJA2‐binding fragment of HDAC2 lay on the N‐terminal domain (residues 322–488) (Figure 4H). Additionally, we utilized reverse Co‐IP assays and found that the RBD domain of PJA2 combined with HDAC2 and the N‐terminal domain of HDAC2 interacted with PJA2 (Figure S7E,F, Supporting Information). Besides, the docking analysis predicted that amino acids 624 and 629 within the RBD domain of PJA2 may contributed to the interaction of amino acids 331333 and 334 within the N‐terminal domain of HDAC2 (Figure 4I; Figure S7G, Supporting Information). Accordingly, specific mutations at the binding sites between PJA2 and HDAC2 were designed (Figure S7H, Supporting Information). These mutations, whether introduced individually to the HDAC2‐binding site or the PJA2‐binding site, or simultaneously to both, effectively reversed the interaction between PJA2 and HDAC2 (Figure 4J). These data verified that HDAC2 is a new substrate of PJA2.

### 2.5 PJA2 Promoted HDAC2 Degradation via the Ubiquitin‐Proteasome Pathway

**Figure 5:**
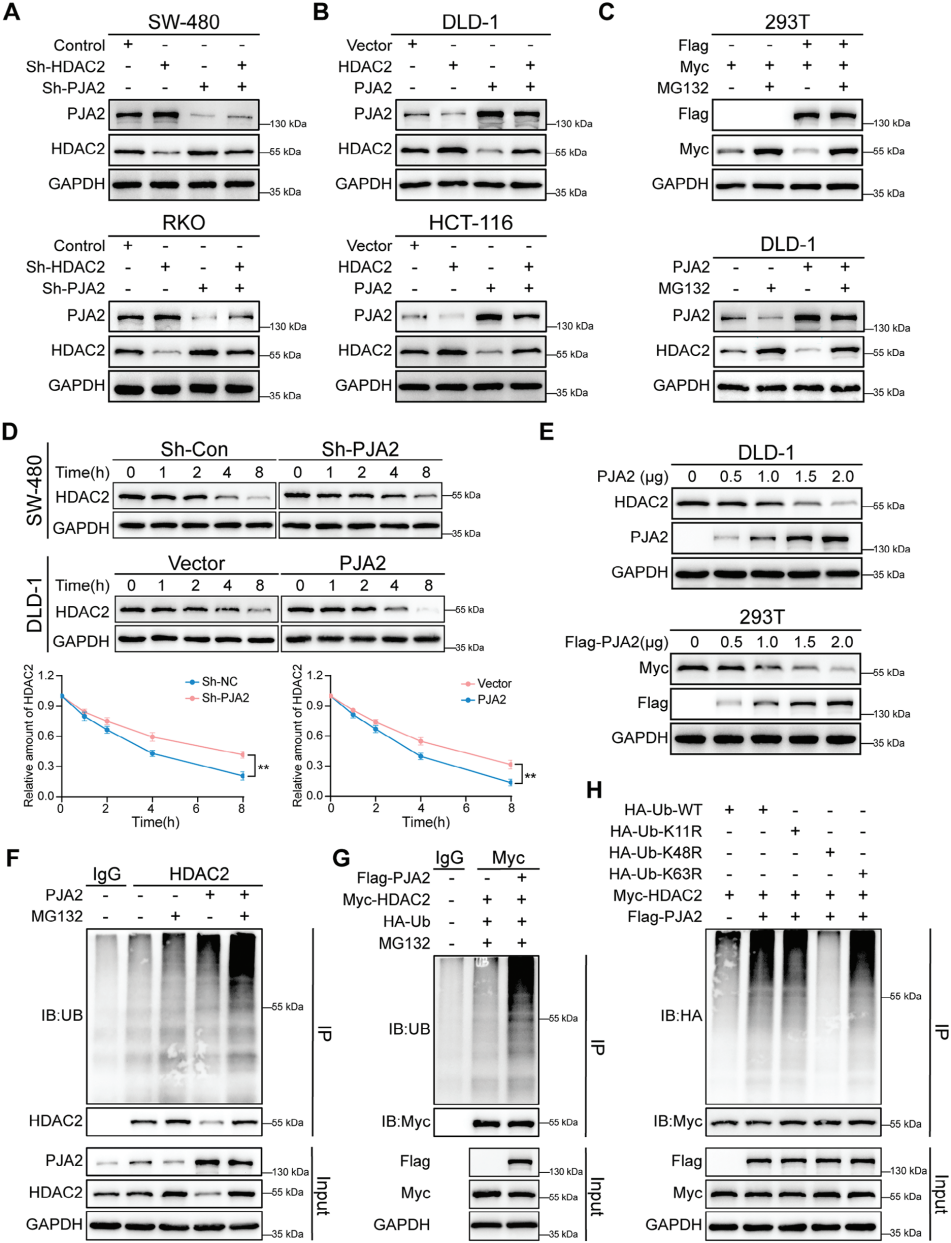
PJA2 promotes HDAC2 degradation through the K48‐dependent ubiquitin‐proteasome way. A,B) Four CRC cells with knockdown or ectopic expression of PJA2 and HDAC2 were collected and then subjected to western blotting. C) The HEK293T cells with overexpression of the indicated Flag‐tagged PJA2 and Myc‐tagged HDAC2 proteins were treated with MG132 for 8 h (top); The DLD‐1 cells with overexpression of the indicated PJA2 protein were treated with MG132 for 8 h (bottom). D) HDAC2 expression levels in SW480 and DLD‐1 cells with the manipulation of PJA2 treated with cycloheximide (CHX) for the indicated times (top) and relative HDAC2 protein levels (bottom). E) DLD‐1 (top) and HEK293T cells (bottom) were transfected with plasmids encoding Myc‐tagged HDAC2 and the indicated amounts of Flag‐tagged PJA2 for 24 h. Cell lysates were analyzed by western blot with indicated antibodies. F) DLD‐1 cells were transfected with Flag‐tagged PJA2. After 48 h transfection, cells were treated with MG132 for 8 h followed by IP and. western blot. G) HEK‐293T cells were transfected with plasmids encoding Flag‐tagged PJA2 and Myc‐tagged HDAC2, along with plasmids encoding HA‐tagged ubiquitin. 24 h after transfection, cells were treated with MG132 for 8 h followed by IP and western blot. H) HEK293T cells were transfected with plasmids encoding Flag‐tagged PJA2 and Myc‐tagged HDAC2, along with plasmids encoding HA‐tagged wild‐type ubiquitin or indicated mutant ubiquitin. 24 h after transfection, cells were treated with MG132 for 8 h. Cell lysates were analyzed by immunoprecipitation with anti‐Myc antibody and western immunoblotting with indicated antibodies. All data are presented as mean ± SD. **p<0.01, ***p<0.001, ****p<0.0001.

Given that PJA2 is an E3 ligase that extensively participates in the degradation of functional proteins like Mob1 and PKA,^[^ ^21^, ^22^ ^]^ HDAC2 was hypothesized to be a new substrate of PJA2. The knockdown and overexpression of PJA2 altered the protein levels of HDAC2 in CRC cells, which could be rescued by manipulating HDAC2 expression (**Figure** **5**A,B), but the expression at mRNA levels of HDAC2 was unaffected (Figure S8A,B, Supporting Information). As shown, treatment with MG132 blocked the ubiquitin‐dependent degradation of HDAC2 protein, suggesting that PJA2 regulates HDAC2 through the ubiquitin‐proteasome pathway (Figure 5C). Besides, PJA2 destabilized HDAC2 in the presence of cycloheximide (CHX) (Figure 5D; Figure S8C,D, Supporting Information), and the ectopic expression of PJA2 inhibited the expression of HDAC2 in a dose‐dependent manner (Figure 5E; Figure S8E, Supporting Information). Consistently, the ubiquitination level of HDAC2 was heightened by PJA2 overexpression and the depletion of PJA2 decreased the ubiquitination level of HDAC2 (Figure 5F,G; Figure S8F, Supporting Information). Moreover, several common ubiquitin mutants were overexpressed in HEK293T cells, and HDAC2 was linked with the K48‐linked poly‐ubiquitination chain (Figure 5H). Together, these results confirmed that PJA2 promoted the K48‐dependent ubiquitination of HDAC2.

### 2.6 Ubiquitination of HDAC2 at K90 Depends on the RING Domain of PJA2

**Figure 6:**
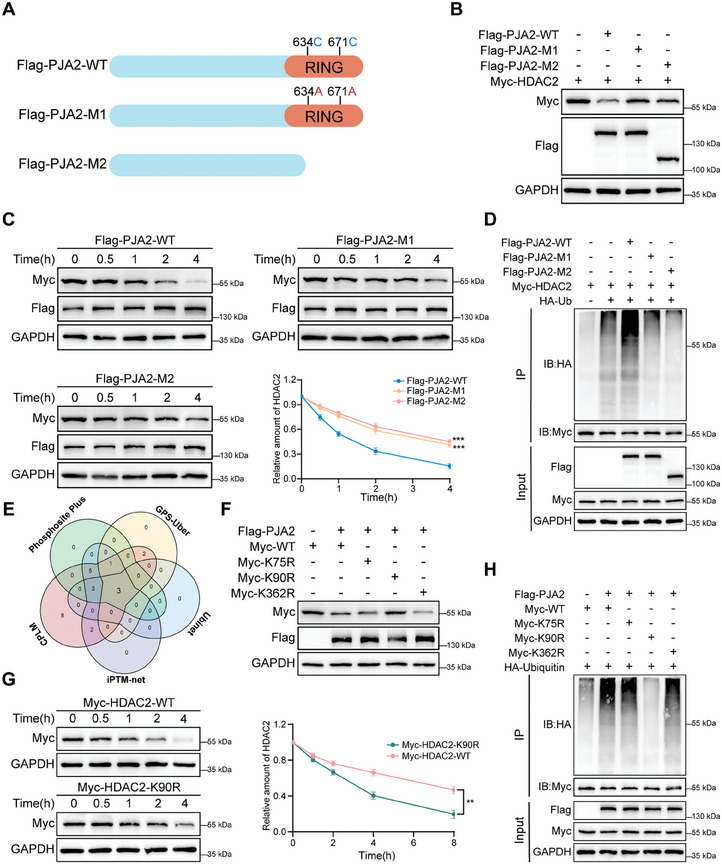
Ubiquitination of HDAC2 at K90 depends on the RING domain of PJA2. A) The schematic diagram of wild‐type and mutants PJA2. B) HEK293T cells were transfected with plasmids encoding Myc‐tagged HDAC2, along with a plasmid encoding Flag‐tagged wild‐type PJA2 or PJA2 mutants (M1, M2). After 24 h, cell lysates were analyzed by western blot with indicated antibodies. C) HEK293T cells were transfected with the indicated plasmids. 24 h after transfection, cells were treated with 100 µg mL^−1^ cycloheximide (CHX) and collected for immunoblot analysis at the indicated time points. And quantification of HDAC2 band intensity was measured by Image J software. D) HEK293T cells were transfected with the indicated plasmids. 24 h after transfection, cells were treated with MG132 for 8 h. Cell lysates were analyzed by IP with anti‐Myc antibody and WB with indicated antibodies. E) The prediction of potential lysine residues. F) Wild‐type and lysine residual mutated Myc‐tagged HDAC2 plasmids were individually transfected into HEK293T cells, with or without Flag‐tagged PJA2. After 24 h, cell lysates were analyzed by western blot with indicated antibodies. G) HEK293T cells were transfected with Myc‐HDAC2‐WT or Myc‐HDAC2‐K90R plasmid for 24 h, and then treated with cycloheximide (CHX) for the indicated times before harvesting. Cell lysates were analyzed by immunoblotting with indicated antibodies (left) and quantification of HDAC2 band intensity was measured by Image J software (right). H) HEK293T cells were transfected with the indicated plasmids for 24 h, followed by treatment with MG132 for 8 h before collection. Cell lysates were subjected to Co‐IP with an anti‐Myc antibody followed by western blotting. All data are presented as mean ± SD. **p<0.01, ***p<0.001, ****p<0.0001.

As a member of the RING finger protein family, PJA2 fulfills its E3 ubiquitin‐protein ligase function via the RING domain (residues 630–708), and the cysteine 634 and 671 are responsible for the enzyme function of PJA2.^[^ ^5^ ^]^ Therefore, Mutant 1 (M1) was constructed by replacing cysteines 634 and 671 with alanines 634 and 671; Mutant 2 (M2) involved the deletion of the RING domain (**Figure** **6**A). Overexpression of wild‐type PJA2 decreased the protein levels of HDAC2, which was not affected when M1 or M2 was overexpressed (Figure 6B). Compared to the shorter half‐life period caused by wild‐type PJA2, the transfection of M1 and M2 prolonged the degradation time of HDAC2 in HEK293T cells (Figure 6C). Further observations also demonstrated that the enhanced ubiquitination of HDAC2 caused by overexpressed PJA2 could be rescued by M1 and M2 (Figure 6D). Collectively, these findings confirm that PJA2 accelerates the degradation of HDAC2 via the RING domain.

Previous studies have reported that HDAC2 can be regulated by ubiquitin ligases in other cancers, while the regulation of HDAC2 in CRC still lacks in‐depth research.^[^ ^23^, ^24^ ^]^ With the help of the three‐dimensional structure of HDAC2 and forecast results from ubiquitination prediction databases (CPLM, Phosposite Plus, GPS‐Uber, Ubinet, and iPTM‐net), three lysine residues were predicted to contribute to the poly‐ubiquitination of HDAC2 (Figure 6E). The overexpression of PJA2 could degrade HDAC2 with wild type, K75R mutant, and K362R mutant, but not the K90R mutant (Figure 6F). Additionally, HDAC2 with the K90R mutant delayed the degradation time, and PJA2 promoted ubiquitination of HDAC2 except in the K90R mutant (Figure 6G,H). Summing up, the RING domain of PJA2 promotes the ubiquitination of HDAC2 at K90 residue.

### 2.7 The PJA2‐HDAC2 Axis Regulates the IFIT Family

**Figure 7:**
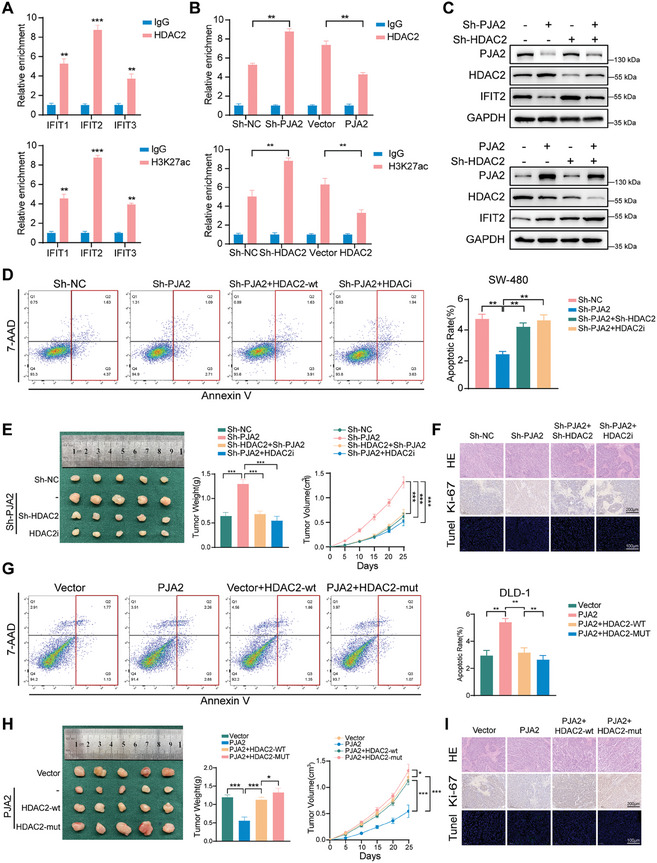
The PJA2‐HDAC2 axis upregulates the transcriptional activity of IFITs. A) ChIP‐qPCR using HDAC2 (top) and H3K27ac (bottom) antibodies to measure their enrichment at the promoter region of selected target genes. B) ChIP‐qPCR using HDAC2 (top) and H3K27ac (bottom) antibodies to measure their enrichment at the promoter region of IFIT2 upon indicated manipulation in SW480 cells. C) Western blot analysis of IFIT2 in SW480 cells stably transfected with the indicated lentiviruses. D) Cells were treated with the serum‐free medium for 36 h. Flow cytometry was used to probe the apoptotic rates (LR+UR) of SW480 cells with indicated treatment. E) Representative photographs of subcutaneous xenograft tumors were obtained from nude mice with indicated treatment. The tumor volumes were measured every five days and the tumor weights were analyzed. F) Representative photographs of H&E, IHC, and Tunel staining in xenograft tumors with indicated treatment. The protein levels of Ki67 and PJA2 in xenograft tumors were detected by IHC. G) Cells were treated with the serum‐free medium for 36 h. Flow cytometry was used to probe the apoptotic rates (LR+UR) of DLD‐1 cells with indicated treatment. H) Representative photographs of subcutaneous xenograft tumors were obtained from nude mice with indicated treatment. The tumor volumes were measured every five days and the tumor weights were analyzed. I) Representative photographs of H&E, IHC, and Tunel staining in xenograft tumors with indicated treatment. The protein levels of Ki67 and PJA2 in xenograft tumors were detected by IHC. All data are shown as the means ± SD of three independent experiments and a *p*‐value under 0.05 was considered to be statistically significant. ns *p* > 0.05, **p < 0.01, ***p < 0.001, ****p<0.0001.

Considering that our RNA‐seq results discovered that overexpressed PJA2 promoted the transcription of the IFIT family and our IP‐MS data showed that PJA2 ubiquitinated and degraded HDAC2, further experiments were performed to examine whether PJA2 regulated the expression of the IFIT family through HDAC2. First, a negative correlation between HDAC2 and the IFIT family was observed in colon adenocarcinoma and rectum adenocarcinoma at the Chipbase database (Figure S4B,D,F, Supporting Information). With the block of HDAC2, the function of PJA2 in regulating the expression at mRNA levels of the IFIT family was abolished (Figure S9A,B, Supporting Information). Considering that acetylation on H3K27 is a hallmark of active enhancers and is regulated by HDAC1 and HDAC2,^[^ ^25^ ^]^ chromatin immunoprecipitation followed by quantitative PCR (ChIP‐qPCR) using HDAC2 and H3K27ac antibodies verified that HDAC2 binds to the promoter of the IFIT family (**Figure** **7**A). Given that IFIT2 was observably upregulated in our results, we chose IFIT2 as the subject of further study. In SW480 cell lines with altered PJA2 and HDAC2 expression, ChIP‐qPCR experiments confirmed that the binding of HDAC2 to the IFIT2 promoter region is regulated by PJA2 expression, and the binding of H3K27ac to the IFIT2 promoter region is regulated by HDAC2 expression (Figure 7B). Furthermore, the knockdown of PJA2 could rescue the increased protein levels of IFIT2 caused by the depletion of HDAC2, while the overexpression of PJA2 could rescue the inhibition of IFIT2 expression by HDAC2 (Figure 7C). Meanwhile, the overexpression of the M1 and M2 mutant forms of PJA2 loses the ability to regulate IFIT2 expression (Figure S9C, Supporting Information). Thus, the PJA2‐HDAC2 axis plays an indispensable role in regulating the transcription of the IFIT family.

### 2.8 HDAC2 Mediates the Tumor‐Suppressing Role of PJA2

To verify the function of the PJA2‐HDAC2 axis in CRC progression, PJA2‐knockdown cells were stably knocked down for HDAC2 or treated with Romidepsin, a potent histone deacetylase inhibitor. In vitro assays showed that the knockdown of HDAC2 and the treatment with Romidepsin rescued the anti‐tumor effect of PJA2 in SW480 and RKO cells (Figure 7D; Figure S9D–G, Supporting Information). Xenograft tumor models showed that the group with HDAC2 knockdown or treatment with Romidepsin suppressed tumor weight and volume compared with tumors with PJA2 knockdown alone (Figure 7E). IHC staining also clarified that these groups rescued the upregulation of Ki‐67 staining and the downregulation of Tunel staining caused by the knockdown of PJA2 (Figure 7F). Furthermore, the overexpression of HDAC2‐mut was established to verify the importance of the combination between PJA2 and HDAC2. In vitro assays exhibited that HDAC2‐mut abolished the tumor suppression caused by PJA2 (Figure 7G; Figure S10A–D, Supporting Information). Moreover, HDAC2‐wt restored tumor proliferation suppressed by PJA2, and HDAC2‐mut promoted tumor growth and inhibited tumor apoptosis compared with HDAC2‐wt in PJA2 overexpressed cells (Figure 7H,I). These results stressed the significance of HDAC2 in the anti‐tumor effect of PJA2.

### 2.9 The Upregulated HDAC2 in CRC can Suppress the Transcriptional Activity of PJA2

**Figure 8:**
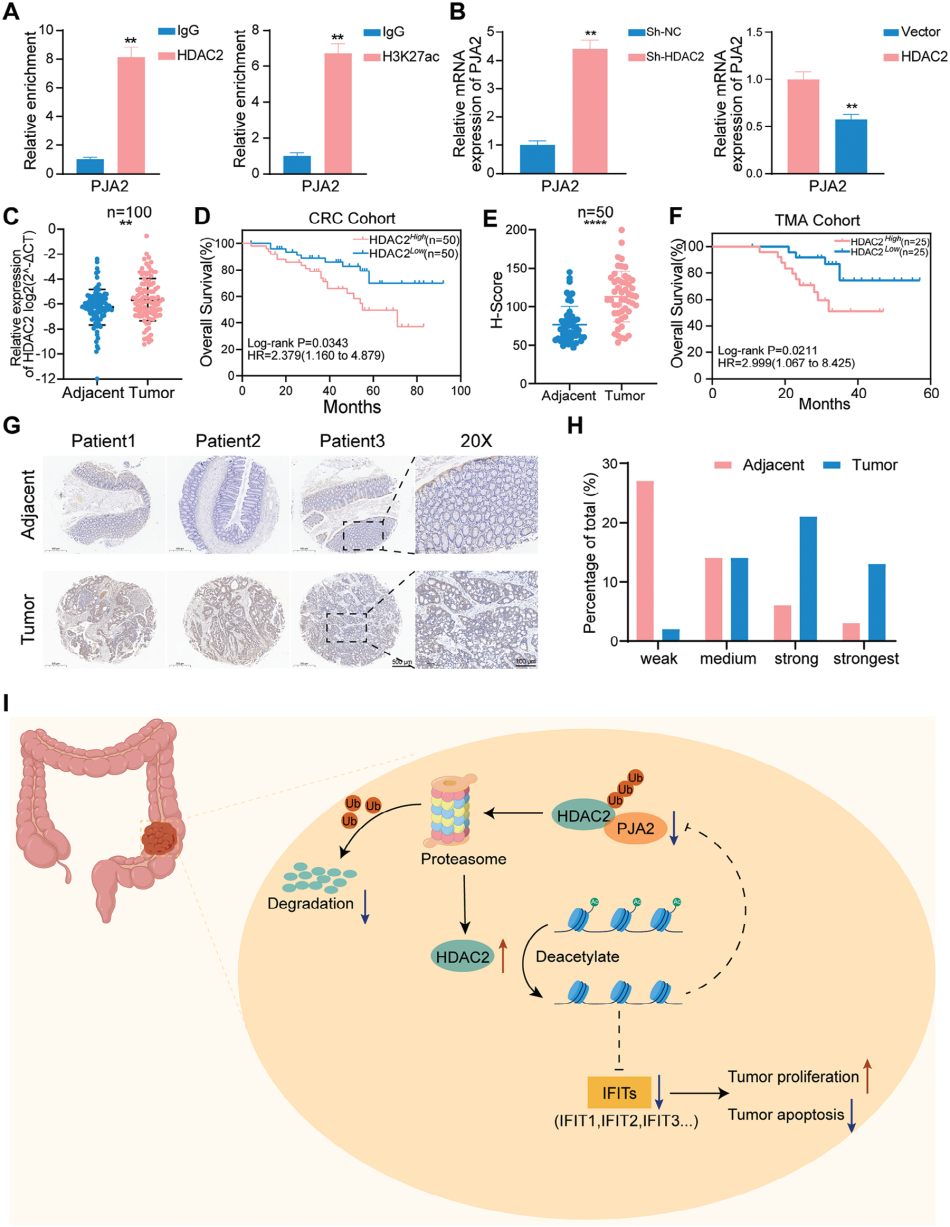
PJA2‐induced HDAC2 protein degradation can suppress the transcriptional activity of PJA2. A) ChIP‐qPCR using HDAC2 (left) and H3K27ac (right) antibodies to measure their enrichment at the promoter region of PJA2. B) The expression at mRNA levels of PJA2 was detected by qRT‐PCR in SW480 cells with the manipulation of HDAC2. C,D) The expression at mRNA levels of HDAC2 C) was detected by qRT‐PCR in 100 paired CRC tissues and adjacent tissues (CRC cohort) and the Kaplan‐Meier OS analysis of HDAC2 expression D). E,F) The H‐score of HDAC2 was detected by IHC in the TMA cohort E), and the Kaplan‐Meier OS analysis of HDAC2 expression F). G,H) The protein level of HDAC2 detected by IHC in the TMA cohort. The representative photographs of HDAC2 expression G), and the relative intensity distribution of IHC H). I) Schematic model of mechanism that PJA2 functioned as a tumor suppressor to regulate colorectal cancer proliferation and apoptosis via ubiquitylation of HDAC2. All data are presented as mean ± SD. **p<0.01, ***p<0.001, ****p<0.0001.

Previous studies have emphasized that HDAC2 inhibits the relaxation of chromatin structure by regulating deacetylation of the histone H3K27ac site, thereby repressing gene transcription^[^ ^26^, ^27^, ^28^ ^]^; similarly, it has been reported that KDM5A‐mediated histone methylation modification could regulate PJA2 transcription, suggesting that histone modification plays an important role in the epigenetic regulation of PJA2.^[^ ^29^ ^]^ Therefore, these studies raise the possibility that PJA2‐mediated degradation of HDAC2 may promote the transcriptional activity of PJA2. Chip assays using HDAC2 and H3K27ac antibodies were performed, and qPCR results verified that HDAC2 could bind to the promoter of PJA2 (**Figure** **8**A). After manipulating HDAC2, the mRNA levels of PJA2 were detected, and the results verified that HDAC2 could inhibit the transcription of PJA2 (Figure 8B). Therefore, the downregulation of HDAC2 resulting from PJA2‐mediated degradation could suppress the transcription of PJA2 and then form a feedback loop to aggravate CRC progression.

HDAC2 has been reported to participate in regulating tumor proliferation and apoptosis, although research about its function in CRC development has been limited.^[^ ^30^, ^31^, ^32^ ^]^ HDAC2 was found to be highly expressed in tumor tissue and patients with higher expression tend to have an unfavorable prognosis (Figure 8C–D). Consistently, the IHC‐score verified that the expression at protein levels of HDAC2 was higher in tumor tissues, and Kaplan‐Meier analysis disclosed a poor prognosis correlated with HDAC2 expression (Figure 8E–H). In the TMA cohort, we observed a negative correlation between PJA2 and HDAC2 expression and a positive correlation between PJA2 and IFIT2 expression (Figure S11A–E, Supporting Information). These findings support the clinical relevance of the PJA2/HDAC2/IFIT2 axis in colorectal cancer samples, suggesting potential diagnostic or prognostic value. All these findings demonstrate that PJA2 interacts with HDAC2 to promote the polyubiquitination and degradation of HDAC2, eliminating the transcriptional inhibition of the IFIT family and PJA2 caused by HDAC2, forming a positive feedback loop and inhibiting tumor proliferation (Figure 8I).

## 3 Discussion

CRC is a cachectic disease in which tumor proliferation significantly contributes to poor prognosis, motivating us to explore the underlying mechanisms driving CRC proliferation. In this context, PJA2 is recognized as a tumor suppressor that can inhibit tumor proliferation and promote apoptosis by restraining HDAC2‐related transcription of the IFIT family. However, this suppression is diminished in patients and mice with CRC, indicating a potential mechanism by which the tumor‐suppressive effects of PJA2 are attenuated in the disease setting.

The current investigation elucidates PJA2 as a pivotal tumor suppressor gene in CRC, adding depth to its established significance in lung adenocarcinoma^[^ ^33^, ^34^ ^]^ and its origin story as an E3 ubiquitin ligase linked to X‐linked mental retardation (MRX).^[^ ^35^ ^]^ Beyond its downregulation in CRC, which positions it as a promising prognostic biomarker, PJA2's demonstrated capacity to restrain tumor proliferation and promote apoptosis underscores its potential as a universal tumor suppressor across a spectrum of malignancies. Particularly in CRC, the protective role of PJA2 against tumorigenesis, as evidenced by our in vitro and in vivo assays and corroborated by the AOM/DSS model, highlights its therapeutic potential. Moreover, PJA2's responsiveness to interferon signaling pathways suggests its involvement in the intricate dance of the immune microenvironment. This responsiveness may enable PJA2 to amplify antitumor immune responses, making it a candidate for integration into immunotherapy strategies. While the exact mechanisms remain to be fully elucidated, the interplay between PJA2 and interferon signaling hints at a novel approach to enhance the efficacy of immunotherapies, particularly in the context of CRC. Thus, PJA2 emerges as a multifaceted target, with implications not only for direct tumor suppression but also for modulating the tumor's interaction with the host's immune system, offering a promising avenue for the development of personalized and combinatorial cancer treatments.

Histone deacetylases (HDACs) are upregulated in various tumors and control the epigenetic programming to regulate cell proliferation and differentiation.^[^ ^36^, ^37^ ^]^ The Class I HDACs, including HDAC1‐3, tend to promote histone deacetylation to inhibit the transcription of target genes.^[^ ^12^, ^38^ ^]^ As IP‐MS data reveals that HDAC2 is a binding partner for PJA2, we focused on the oncogenic role of HDAC2 in CRC. Previous studies disclosed that HDAC2 could promote metastasis of pancreatic cancer by restraining the expression of E‐cadherin.^[^ ^39^ ^]^ HDAC2 is necessary for the anti‐apoptosis effects in CRC and is greatly upregulated with the loss of APC, emphasizing its role in tumorigenesis.^[^ ^14^ ^]^ Furthermore, emerging research demonstrates that the block of HDAC2 can accelerate tumor apoptosis and restrain cancer development.^[^ ^40^, ^41^, ^42^ ^]^ We identify that the N‐terminal domain of HDAC2 physically combined with the RBD domain of PJA2 and that PJA2‐mediated ubiquitination depends on the K90 residue of HDAC2 and the RING domain of PJA2. With the loss of acetylation, the decreasing transcriptional activity of the IFIT family indicates that HDAC2 mediates the carcinogenic effects of PJA2. In CRC tissues, the upregulated HDAC2 due to the reduction of PJA2 restrains the transcription of PJA2 and forms a feedback loop to aggravate tumor progression (Figure 8I). Given the importance of HDACs and E3 ligases in cancer biology, the proposed feedback loop involving HDAC2 and PJA2 may represent a common regulatory mechanism in other cancers. This mechanism could contribute to the development and progression of various malignancies by altering the balance of gene expression and cellular signaling pathways. Understanding the intricate interplay between HDAC2 and PJA2 offers a promising avenue for the development of targeted therapies aimed at restoring balance and enhancing the efficacy of current treatments, particularly in the context of CRC. This feedback loop may serve as a biomarker for prognosis and a therapeutic target, opening new horizons for personalized medicine in oncology.

Beyond the scope of our current findings, several unexplored aspects warrant further investigation. First, while our study primarily verifies that PJA2 can regulate tumor proliferation and apoptosis, the role of PJA2 in other malignant phenotypes such as invasion and migration remains unknown. Secondly, we have focused on exploring the role and potential mechanisms of the PJA2/HDAC2 axis in colorectal cancer cells, but we recognize that further investigation into its effects in other tumor types is warranted. For example, single‐cell sequencing datasets for gastric cancer (HRA001689) reveal expression trends of the PJA2/HDAC2 axis that parallel those observed in colorectal cancer datasets (HRA000201). Therefore, the study of this axis in a pan‐cancer context necessitates additional efforts in our future research. Thirdly, considering the potential of HDAC inhibitors as a chemotherapeutic regimen for colorectal cancer, their efficacy and safety require further investigation. The focus on the PJA2‐HDAC2 axis, which forms a feedback loop, highlights the critical role of HDAC inhibitors in modulating HDAC2‐mediated histone deacetylation. Given this, the interplay between HDAC inhibitors and PJA2 represents a highly valuable research direction that warrants further exploration. These issues remain to be addressed, and we plan to solve them in our future studies.

Collectively, our findings demonstrate that PJA2 mediates K48‐dependent ubiquitination and degradation of HDAC2, leading to increased transcriptional activity of the IFIT family. The downregulated HDAC2, in turn, restrains the transcription of PJA2, forming a feedback loop that enhances the tumor‐inhibitory effect of PJA2 (Figure 8I). These results collectively suggest that PJA2 could serve as a prognostic marker and warrant further clinical translational research to explore its therapeutic potential in colorectal cancer.

## 4 Experimental Section

### Human Specimens and Cell Lines

All 100 samples came from the First Affiliated Hospital of Nanjing Medical University and were stored at −80 °C for preservation. All patients had informed consent and none of them had preoperative chemoradiotherapy. The tissue microarray (TMA) with 50 paired patients was made by Servicebio (Wuhan, China). The clinicopathological information of these patients is listed in Tables S2 and S3 (Supporting Information). The Ethics office of the First Affiliated Hospital of Nanjing Medical University approved our research.

The mentioned cell lines (SW480, RKO, DLD‐1, LoVo, SW620, Caco2, HT‐29, HCT116, and HEK‐293T) were acquired from the Shanghai Institute of Cell Biology. All cells were cultured in the recommended culture medium (Gbico) with 10% FBS.

### Western Blot (WB) Analysis and Antibodies

WB was implemented as previously reported.^[^ ^43^ ^]^ The primary antibodies consumed are listed in Table S4 (Supporting Information).

### RNA Isolation and Quantitative Real‑Time PCR (qRT‑PCR)

RNAiso Reagent (TaKaRa) was used to isolate total RNA and qRT‐PCR was performed according to previous procedures.^[^ ^44^ ^]^ The primer sequences are listed in Table S5 (Supporting Information).

### Plasmid Construction and Cell Transfection

The lentivirus (LV) including short hairpin RNAs (shRNAs) targeting PJA2 and HDAC2 was designed and acquired from Genomeditech (Shanghai, China). The full length of PJA2 and HDAC2 synthesized by Genomeditech (Shanghai, China) were transfected into lentivirus vectors.

PJA2 overexpression plasmid and shRNAs plasmids, HDAC2 overexpression plasmid and shRNAs plasmids, and ubiquitin overexpression plasmid were designed and acquired from Genomeditech (Shanghai, China). The mutant expression plasmids of PJA2 with a FLAG tag and of HDAC2 with a Myc tag were synthesized by Genomeditech (Shanghai, China). Lipofectamine 3000 (Invitrogen) was utilized as a transfection tool.

### Cell Proliferation Assays

We used Cell Counting Kit‐8 (Beyotime) to detect cell proliferation as in previous descriptions.^[^ ^44^ ^]^ Briefly, we inoculated 1 × 10^3^ cells into 96‐well plates and cultured them for 1d, 2d, 3d, 4d, and 5d. The absorbance at 450 nm was then determined by the enzyme immunoassay analyzer (Thermo Fisher Scientific).

For the colony formation experiment, as previously reported, we planted the treated cells on a six‐well plate and stained them with crystal violet two weeks later.^[^ ^44^ ^]^

As mentioned before, the EdU assay kit (Beyotime) was used to detect cell proliferation.^[^ ^44^ ^]^ 1 × 10^3^ cells were inoculated on the 96‐well plate for 24h, and follow‐up experiments were conducted according to the kit instructions.

### Flow Cytometry Assays of Cell Apoptosis

The proportion of apoptotic rate was detected by CytoFLEX Flow Cytometer (Beckman). The experimental procedure followed the previous descriptions.^[^ ^45^ ^]^ After treatment, the cells were cultured in a serum‐free medium for 36 h to induce apoptosis. Apoptosis was detected using Annexin V‐APC /7‐AAD apoptosis kit (Multi‐Science). The treated cells were digested and stained with AnnexinV‐APC and 7‐AAD staining solutions according to the instructions.

### RNA Sequencing Assay (RNA‑seq)

The RNA‐sequencing analysis of Vector‐SW480 and PJA2‐SW480 was operated following previous descriptions.^[^ ^45^ ^]^ The processing and analysis of sequencing data were conducted by SEQHEALTH (Wuhan, China). Briefly, we used the DESeq2 package to identify DEGs with FDR < 0.05 as a screening criterion. We used the KOBAS tool to conduct GO enrichment analysis for DEGs with significantly up‐regulated or down‐regulated expression, and the GSEA tool to conduct GSEA enrichment analysis for DEGs with differentially expressed expression. The raw data is available in GEO under the number GSE247535.

### Co‐Immunoprecipitation (Co‐IP) and GST Pull‐Down Assays

The physical combination between PJA2 and HDAC2 was detected by Pierce Classic Magnetic IP/Co‐IP Kit (Thermo Fisher Scientific). The cell lysate was incubated with 5ug antibody at 4 °C rotation overnight. The immunocomplex was incubated with A/G magnetic beads for 1h with rotation. After washing 3 times with IP buffer and pure water, the immunoprecipitated proteins separated from magnetic beads were mixed with 1x SDS loading buffer and boiled for 10 min. Further Western blot and Mass spectrometry (BGI, Shenzhen, China) were utilized for verification.

After GST‐PJA2 and Myc‐HDAC2 plasmids were transfected into bacteria BL21 (*E. coli*), fusion proteins were separated and purified according to the protocol of Thermo Fisher Scientific. Glutathione agarose beads were incubated with purified protein for 12 h, and the interacting protein was washed 3 times and eluted with elution buffer. Following the manufacturer's instructions, the protein lysate collected by GST Pull‐down was separated on SDS‐PAGE and analyzed by Coomassie Brilliant Blue (Beyotime) staining or Western blot with the anti‐GST and anti‐Myc antibodies.

### In Vitro Ubiquitination Assay

In cells that have been transfected with specific plasmids, hemagglutinin (HA)‐tagged ubiquitin (Genomeditech) was transiently transfected and MG132 (Beyotime) was used 8 h before protein extraction. Then, the lysates were used for immunoprecipitation with an anti‐Myc antibody, and ubiquitination levels were analyzed by WB.

### Chromatin Immunoprecipitation Assays (CHIP)

The Sonication Chromatin IP Kit (CST, #56 383) was utilized to perform the ChIP assay, according to the procedure previously reported.^[^ ^46^ ^]^ In brief, about 4 million cells were prepared and chromatin fragments were extracted with the ultrasonic treatment. The lysate was incubated with corresponding antibodies and magnetic beads. After the purification and extraction of DNA, qRT‐PCR was used to analyze the ChIP results. The ChIP primer sequences are listed in Table S5 (Supporting Information).

### Immunofluorescence (IF)

The immunofluorescence assay was conducted as previously described.^[^ ^45^ ^]^ The primary antibodies and fluorescent secondary antibodies are listed in Table S4 (Supporting Information).

### Immunohistochemistry (IHC)

The Immunohistochemistry assay was constructed by Servicebio (Wuhan, China) as the previous description.^[^ ^45^ ^]^ The analysis data of IHC, including the percentage of positively stained cells, the staining intensity, and the histochemistry score (H‐score), was provided by Servicebio.^[^ ^45^ ^]^

### Proximity Ligation Assay (PLA)

SW480 and DLD‐1 cells were cultured and seeded onto confocal dishes, allowing them to adhere overnight. Cells were fixed with 4% paraformaldehyde for 60 min at 37 °C, permeabilized, and blocked. Primary antibodies against PJA2 and HDAC2 were incubated overnight at 4 °C. The next day, cells were processed according to the manufacturer's instructions for the NavinciFlex Cell MR Kit (Navinci, Atto647N), including PLA probe incubation, ligation, and amplification steps. Cell nuclei were stained with DAPI, and PLA signals were visualized using a Thunder Imager rapid high‐resolution inverted fluorescence microscope (Leica).

### Animal Models

Six‐week‐old BALB/c nude mice and C57/B6 mice were acquired from the Animal Core Facility of Nanjing Medical University. The animal experimental ethics number of our study is IACUC‐2306044.

For subcutaneous models, 1×10^6^ colorectal cancer cells stably transfected with shControl, shPJA2, Vector, and PJA2, and their mutants were subcutaneously injected into the armpits of the mouse limbs. As tumors grew up to 100 mm^3^, 1 µg IFNγ was intratumorally injected twice a week and 1mg kg^−1^ Romidepsin was intratumorally injected thrice a week.^[^ ^47^, ^48^ ^]^ Tumors were measured and calculated every five days in the described formula mode: tumor volume = (length × width^2^)/2.^[^ ^49^ ^]^ All mice were sacrificed 25 days after the injection, and further IHC staining was applied.

For AOM/DSS colorectal tumorigenesis models, the procedure of this animal model was previously expounded.^[^ ^49^ ^]^ Briefly speaking, Adeno‐associated virus (AAV) was intraperitoneally injected two weeks before CAC induction to alter the expression of PJA2. C57/B6 mice were intraperitoneally injected with 10mg kg^−1^ azoxymethane (AOM, Sigma) and drank normal water for 1 week. Then 2.5% dextran sulfate sodium (DSS, MP Biologicals) was provided for 1 week, and normal water was provided for 2 weeks. This progression was repeated in three cycles. In the last two cycles, 20ug interferon or PBS was intraperitoneally administered to mice once a week.^[^ ^47^, ^48^ ^]^ After the completion of three modeling cycles and following an approximate 3‐week post‐modeling waiting period, all mice were euthanized, and their colons were excised for further analysis.

All animal experiments were conducted in accordance with the National Institutes of Health guidelines for the use of laboratory animals.

### Statistical Analysis

All statistical analyses were operated by GraphPad Prism 9.0 and shown as means ± standard deviation (SD). Statistically significant variables found in univariate analysis were entered into logistic regression analysis to identify the independent risk factors of CRC. Comparison of survival rates between different groups was computed by the Kaplan–Meier method and log‐rank test. Comparisons between groups were made by utilizing the Student's *t*‐test and Chi‐square test. *p* < 0.05 was considered to reach statistical significance.

### Ethics Approval and Consent to Participate

The committee on the ethics of The First School of Clinical Medicine, Nanjing Medical University, approves human tissue study. The ethics permission number is 2019‐SRFA‐131. All animal experiments are conducted with the approval of the Committee on the Ethics of Animal Experiments of Nanjing Medical University. The ethical number of the animal experiment is IACUC‐2306044.

## Conflict of Interest

The authors declare no conflict of interest.

## Author Contributions

Z.C., C.P., T.W., C.J., and Y.W. contributed equally to this work. Y.S., Z.C., Y.F., and J.T. generated the hypothesis and designed the experiments; Z.C., P.Y., C.J., T.W., and Y.W. performed experiments; X.W., C.P., Q.S., H.X., and H.N. helped to interpret the data; Z.C. wrote the manuscript; Y.S. supervised the overall research, secured funding, and interpreted results. All authors read and approved the final manuscript.

## Supporting information



Supporting Information

Supporting Information

## Data Availability

The data that support the findings of this study are available from the corresponding author upon reasonable request.
