# Supplementary material for: PJA2 Suppresses Colorectal Cancer Progression by Controlling HDAC2 Degradation and Stability
Source: Adv Sci (Weinh). 2025 Feb 10;12(13):2401964. doi: 10.1002/advs.202401964 (PMC11967759; doi:10.1002/advs.202401964)
Supplement: Supplementary file 1 — Supporting Information [file ADVS-12-2401964-s002.docx]

**Supplementary Figures**

**Figure S1**


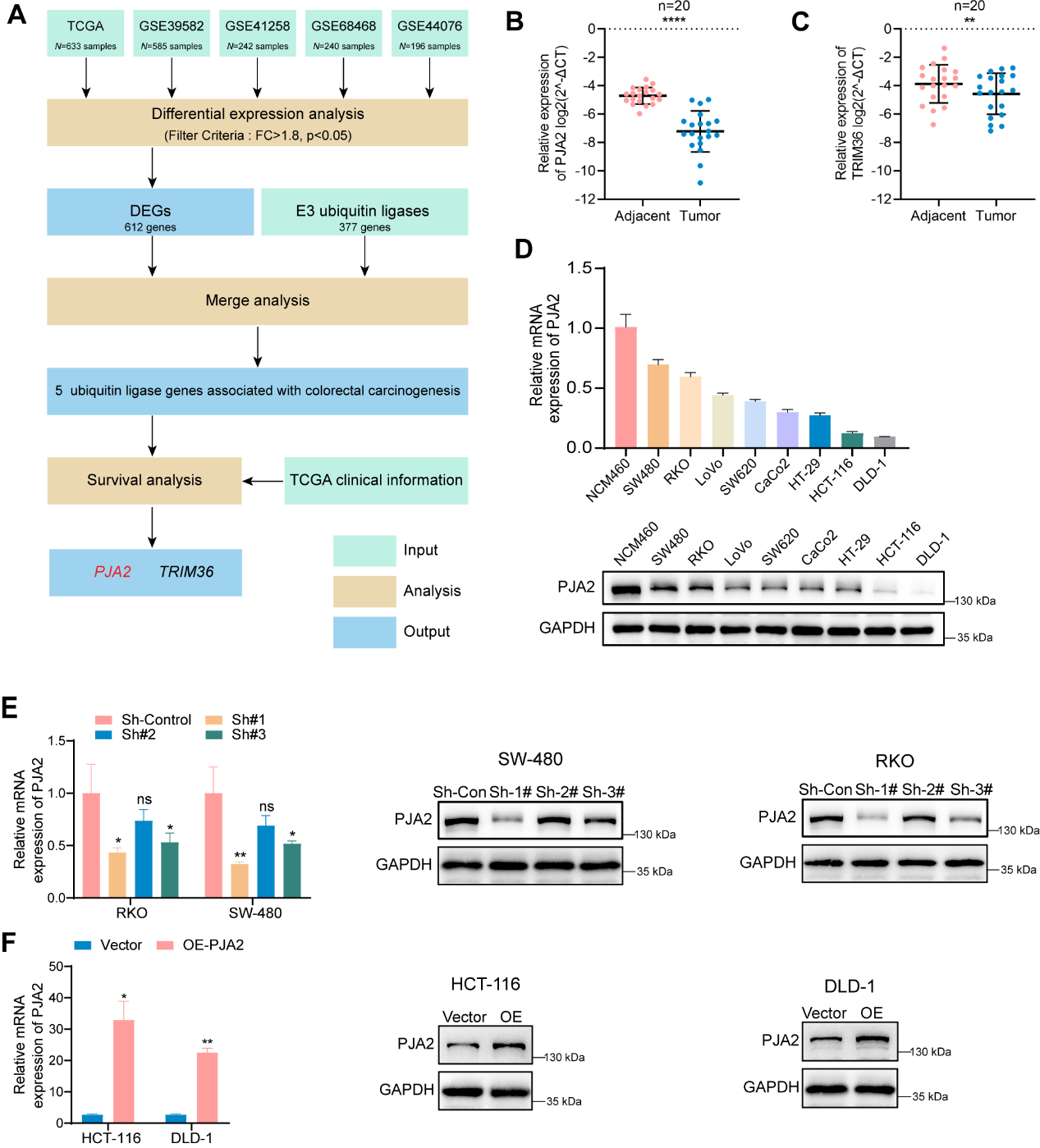


**Figure S2**


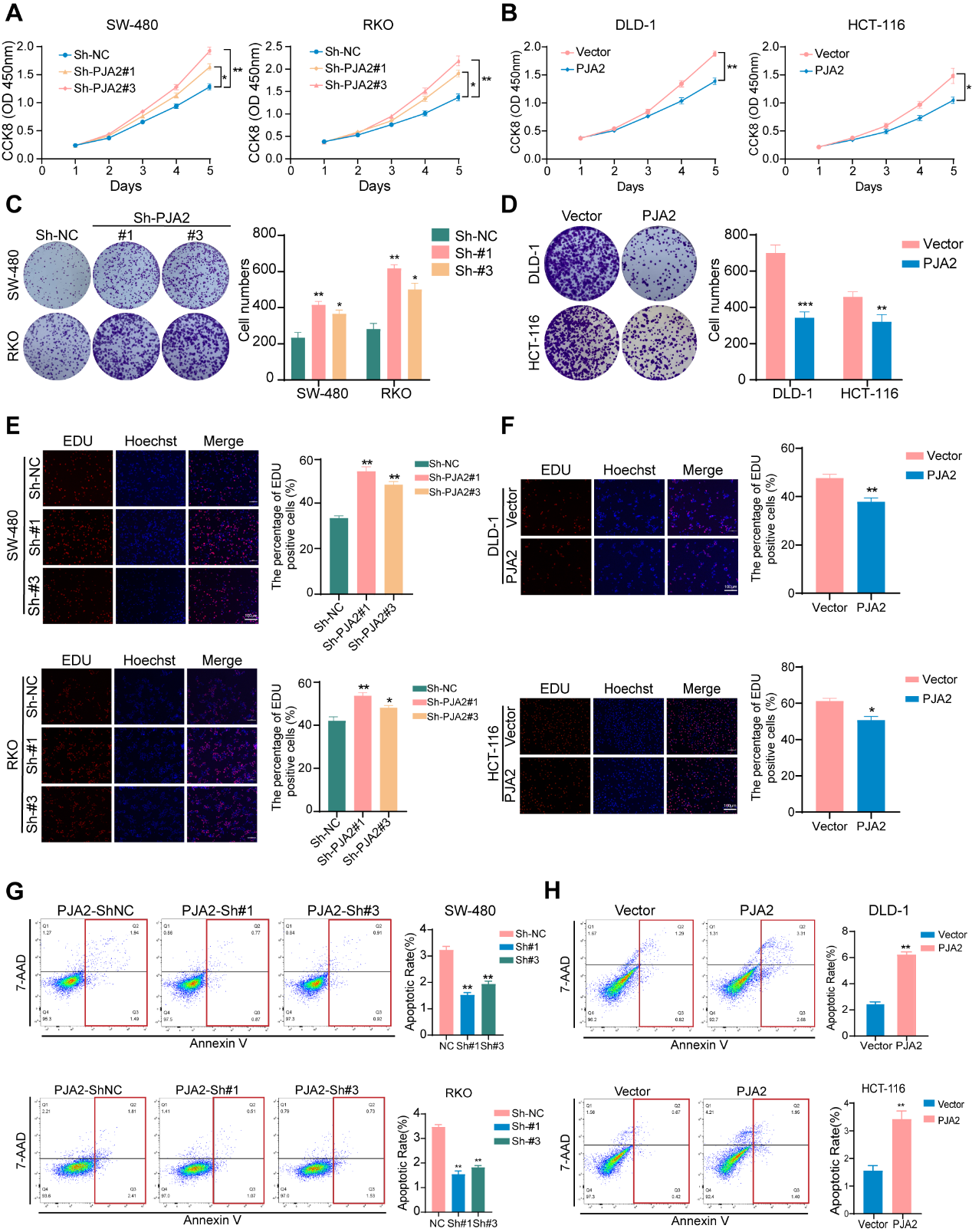


**Figure S3**

**
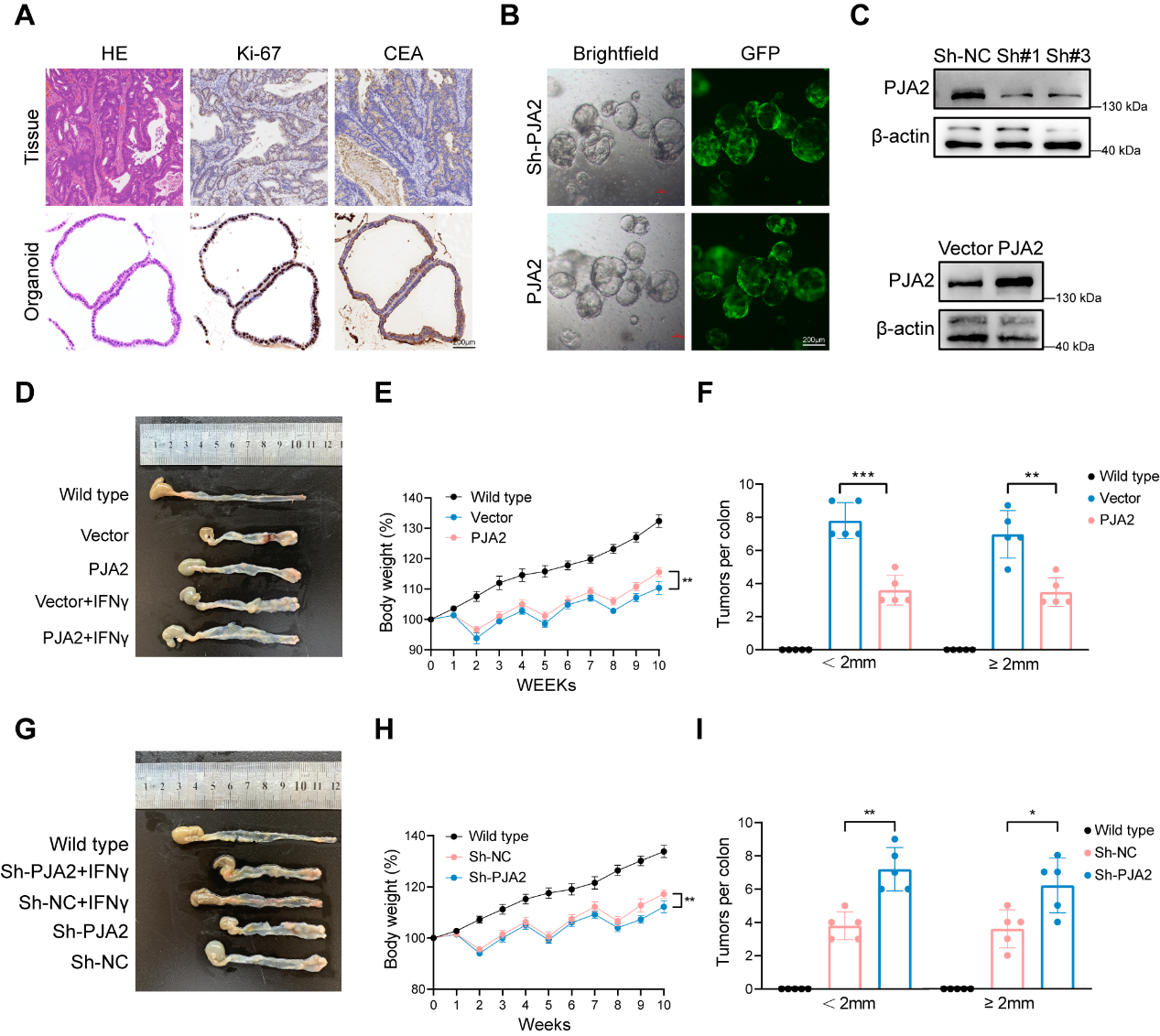
**

**Figure S4**
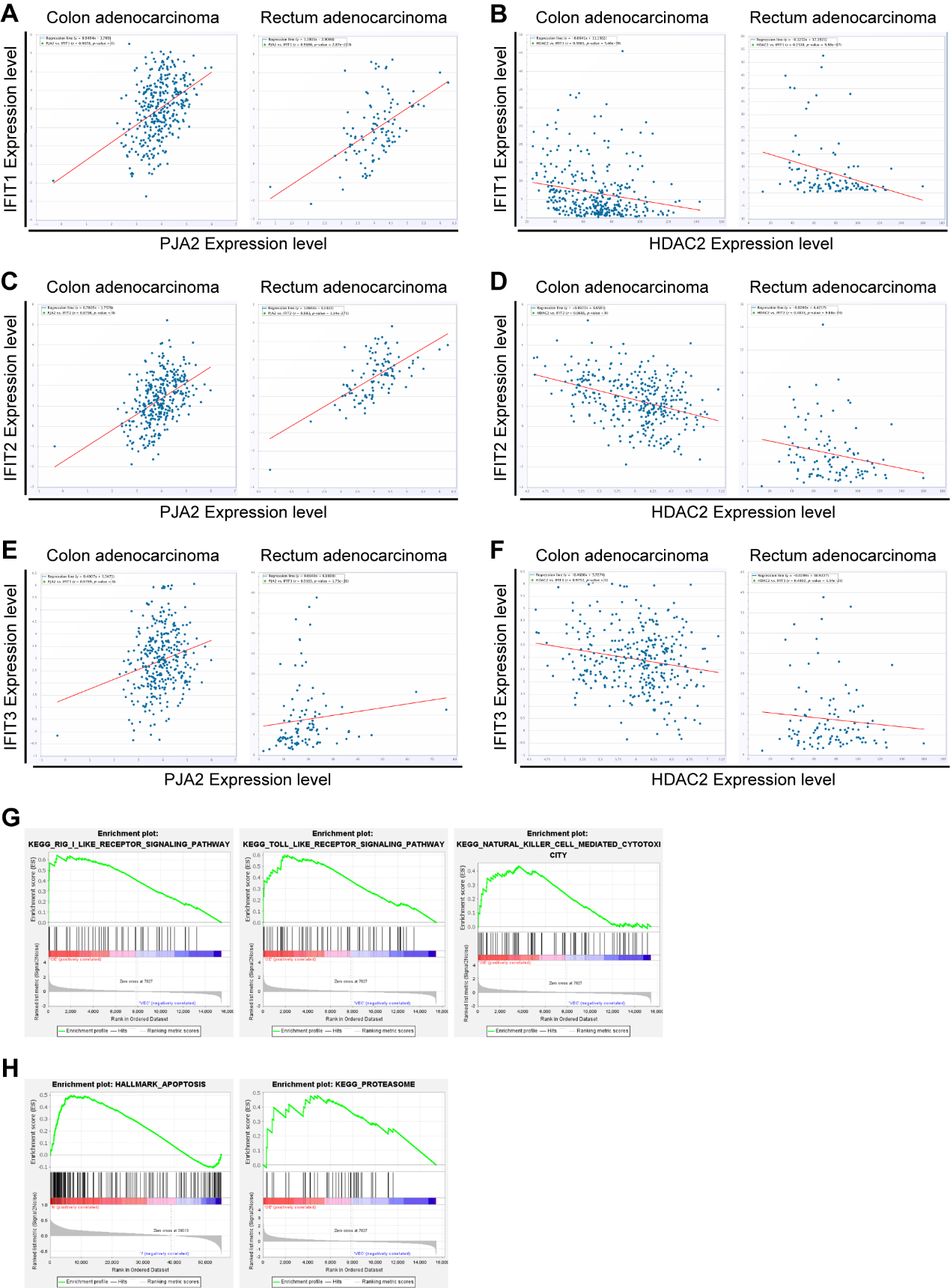


**Figure S5
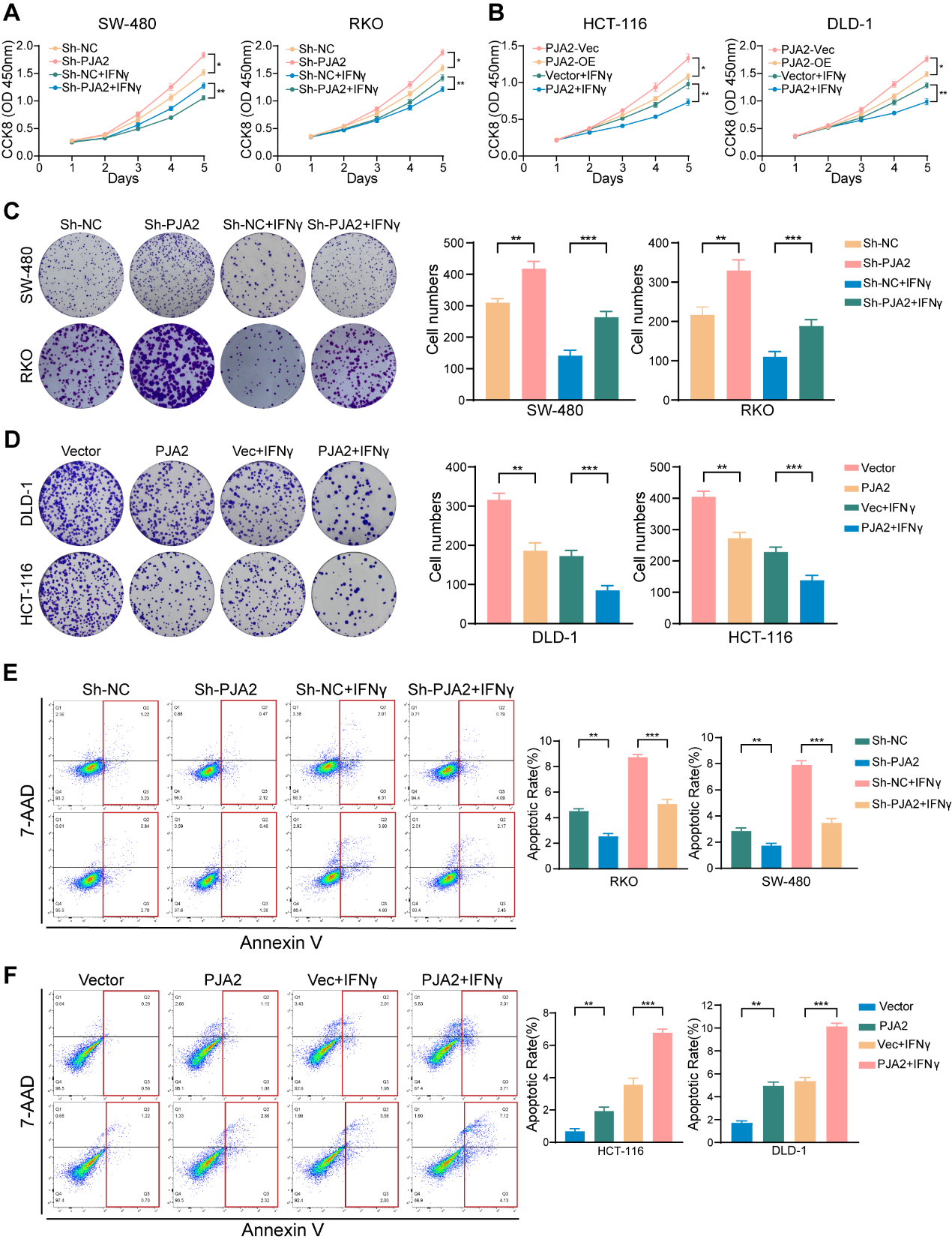
**

**Figure S6**
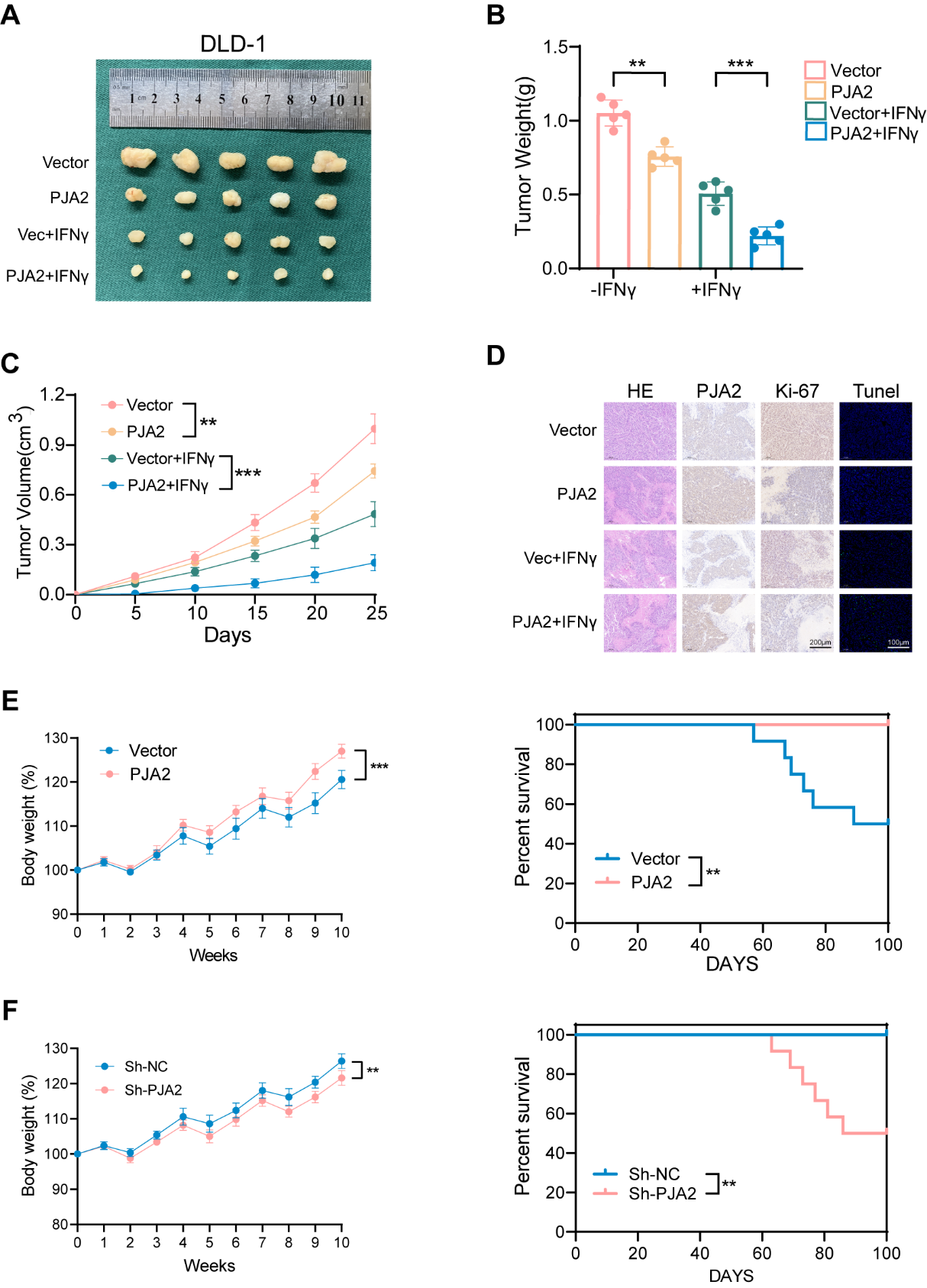


**Figure S7**
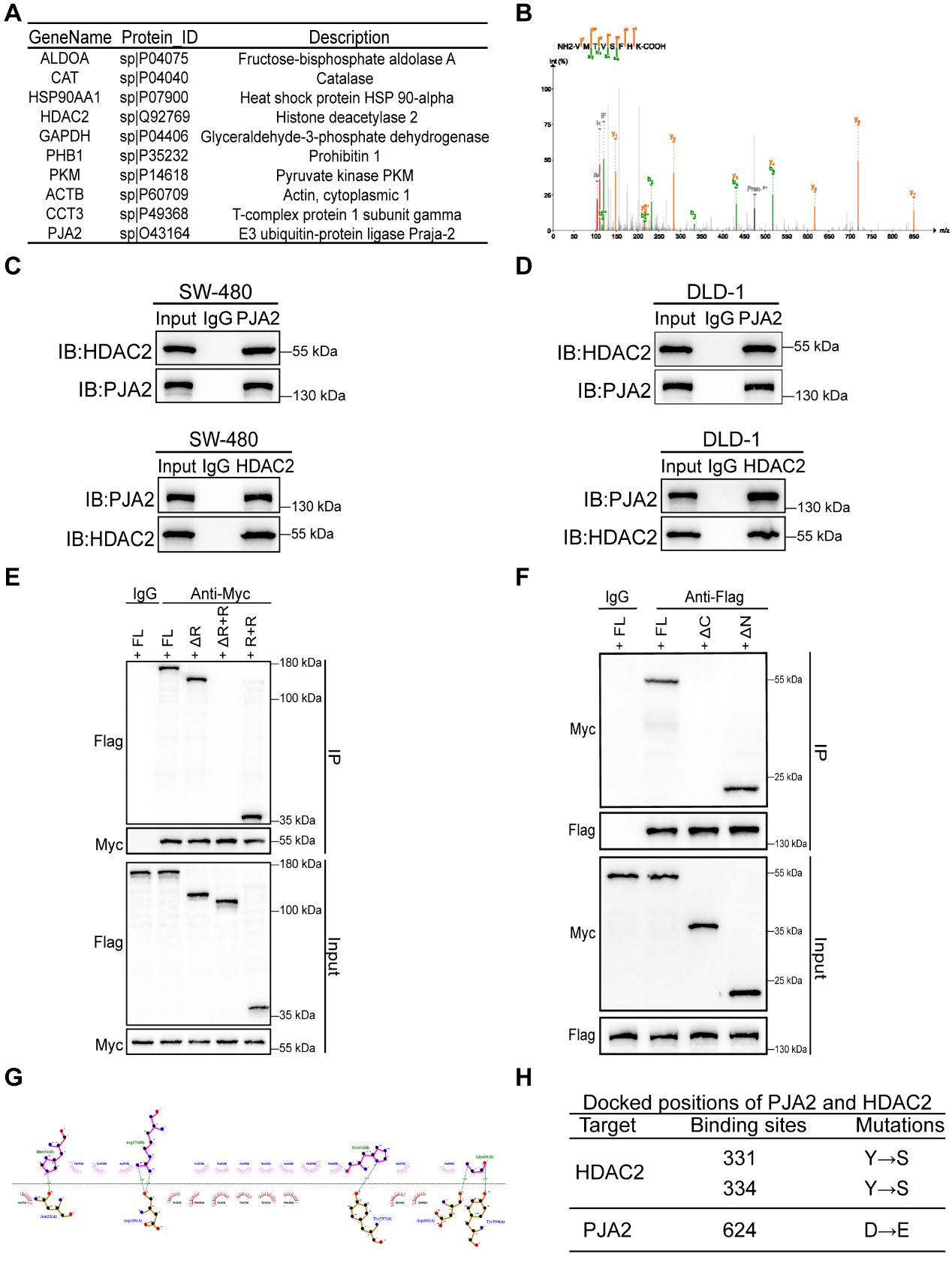


**Figure S8**
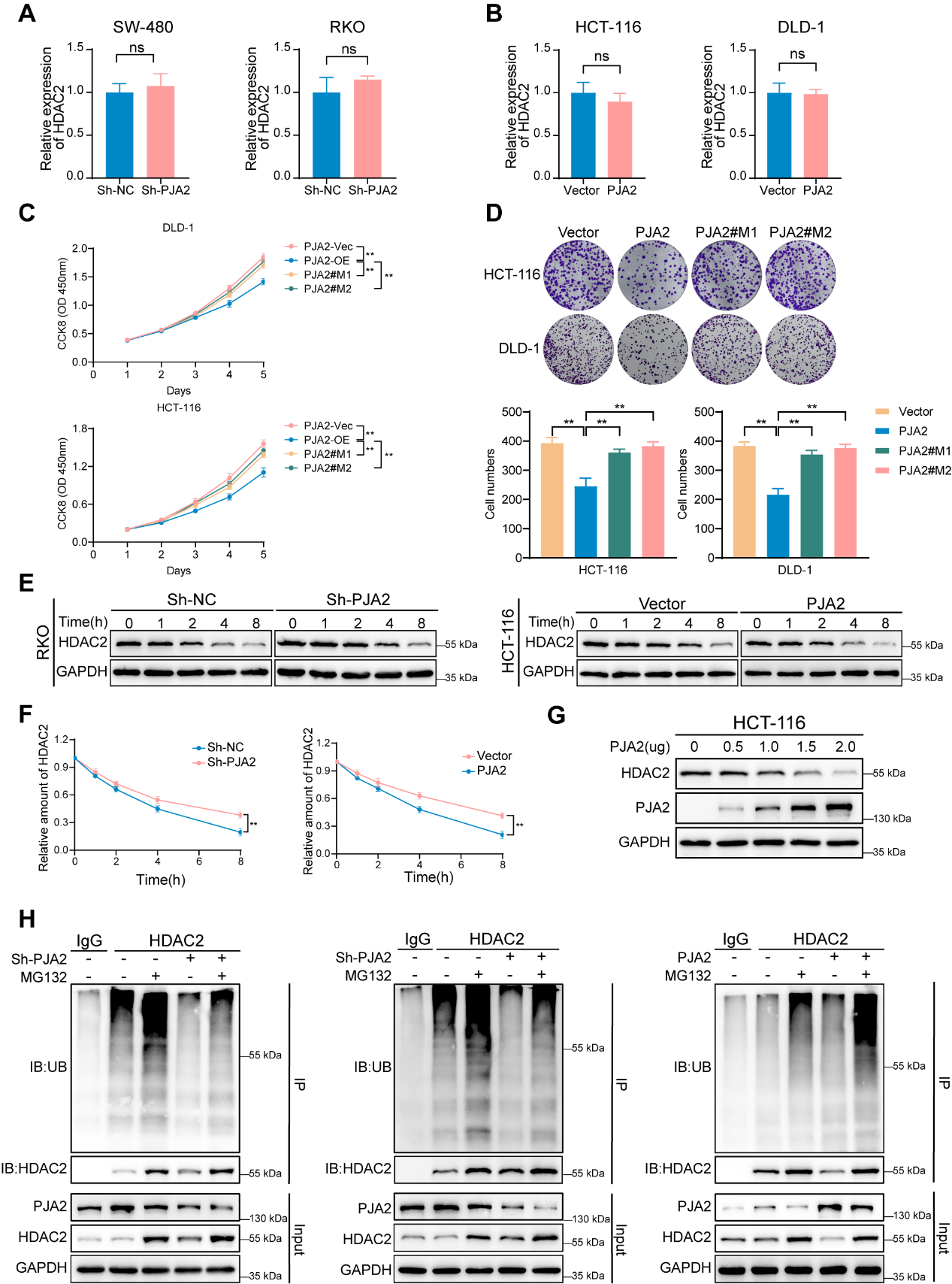


**Figure S9**
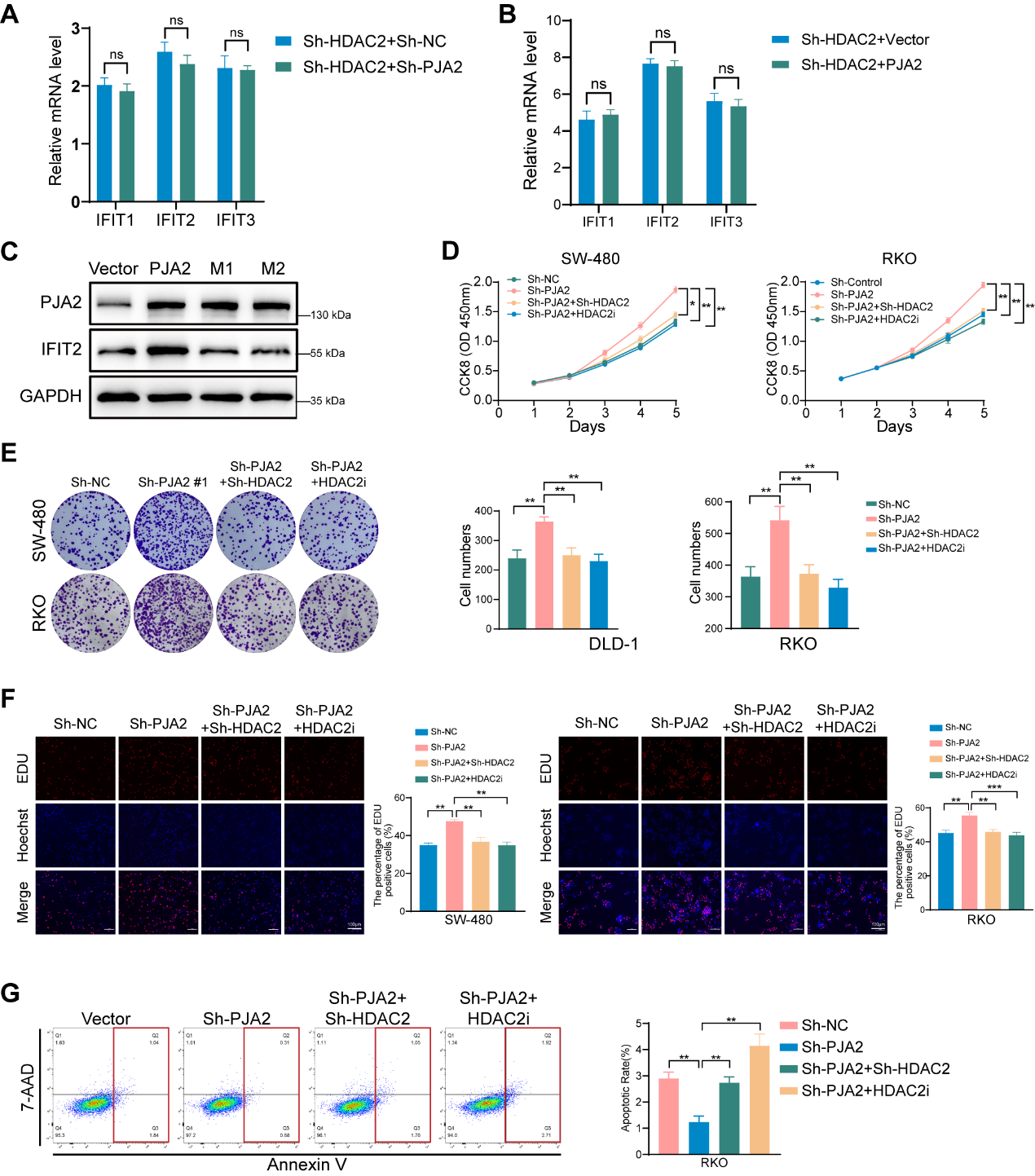


**Figure S10**
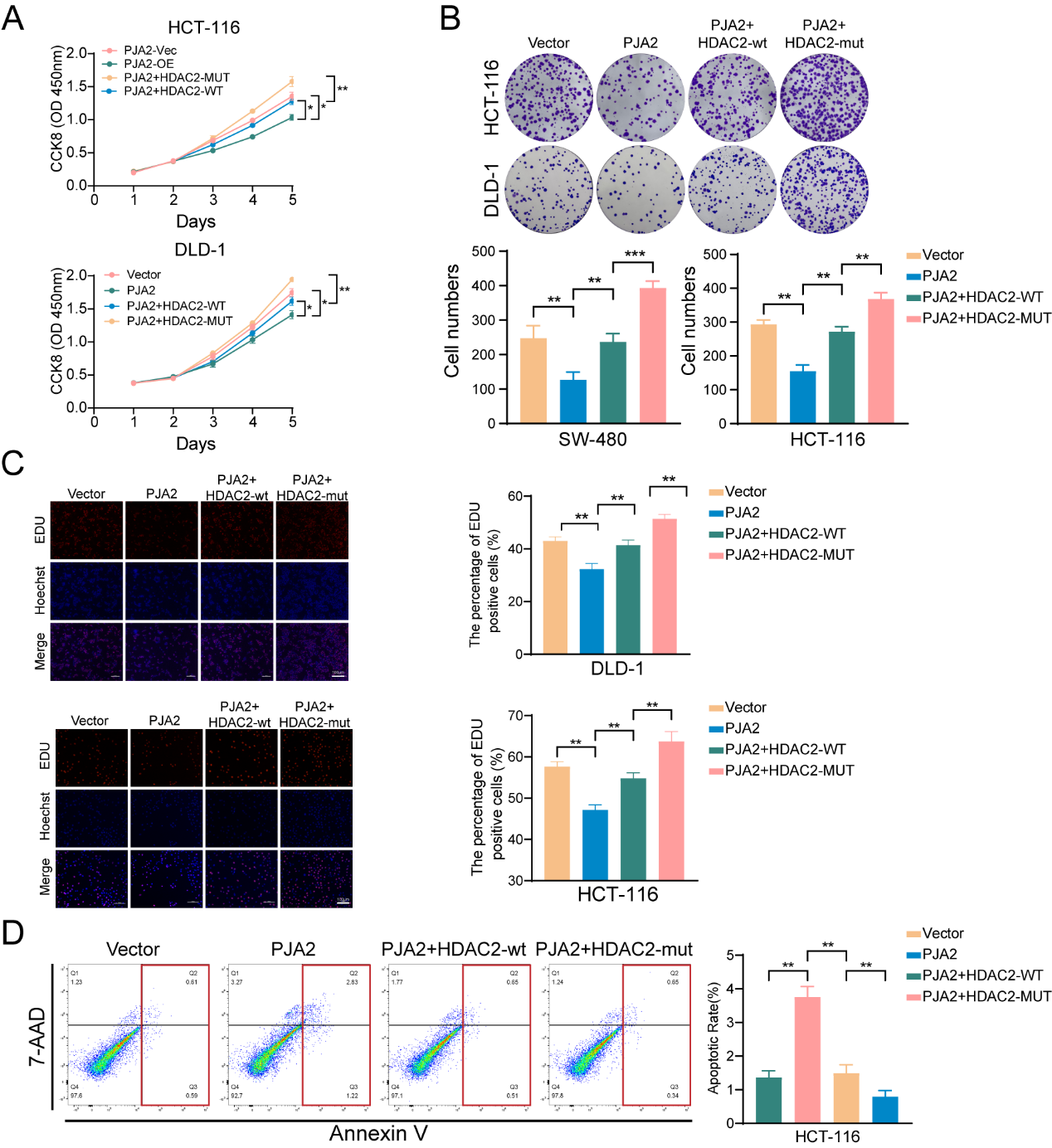


**Figure S11**
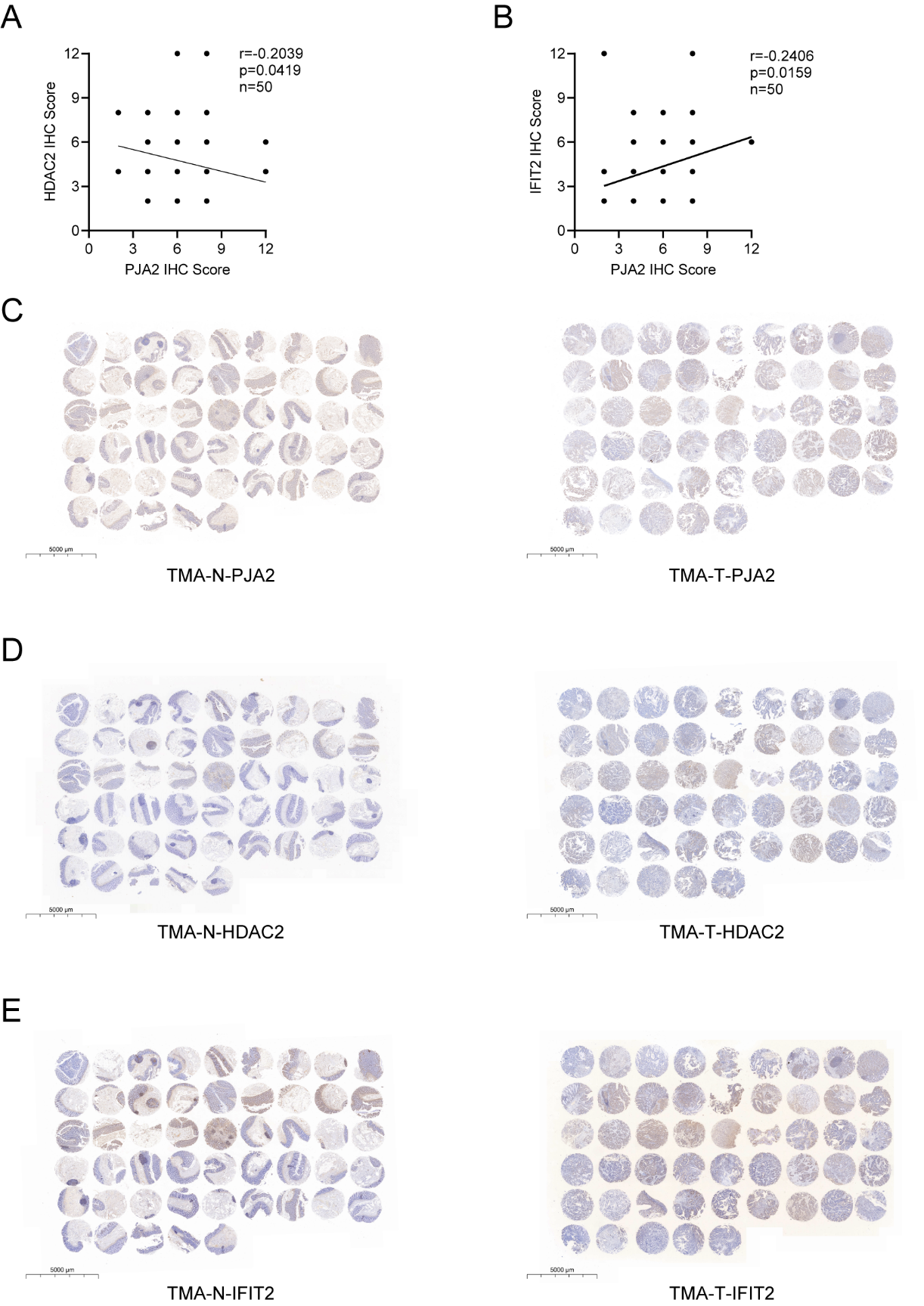


**Supplementary Figure legends**

**Figure S1. The expression of PJA2 in public datasets and CRC cells. A.** Flowchart displaying the procedure used to identify candidate genes. **B.** The mRNA expression of PJA2 was detected by qRT-PCR in 20 paired CRC tissues and adjacent tissues, with GAPDH as the internal reference gene. **C.** The mRNA expression of TRIM36 was detected by qRT-PCR in 20 paired CRC tissues and adjacent tissues, with GAPDH as the internal reference gene. **D.** Relative expression of PJA2 was detected by qRT-PCR (upper) and western blot (lower) in CRC cell lines. **E.** The knockdown efficiency of PJA2 in SW480 and RKO cells was analysed by qRT-PCR (left) and western blot (right). **F.** The overexpression efficiency of PJA2 in HCT116 and DLD-1 cells was analysed by qRT-PCR (left) and western blot (right). All data are shown as the means ± SD of three independent experiments and a P-value under 0.05 was considered to be statistically significant. ns P > 0.05, **P < 0.01, ***P < 0.001, ****P<0.0001.

**Figure S2. PJA2 suppresses proliferation and promotes apoptosis of CRC cells *in vitro*. A-B.** CCK8 assays were applied to evaluate the viability of PJA2 knockdown or overexpression cells. **C-D.** Colony formation assays were used to detect cell proliferation ability. **E-F.** EdU staining assays were conducted to assess the evaluate the

cell proliferation ability. **G-H.** PJA2 knockdown cells (**G**) and PJA2 overexpression cells (**H**) were treated with serum-free medium for 36 hours, and Flow cytometry was used to probe the apoptotic rates (LR+UR) of cells. Data are shown as mean±SD of three independent experiments, *P<0.05, **P<0.01, ***P<0.001, ****P<0.0001.

**Figure S3. PJA2 suppresses proliferation and promotes apoptosis of CRC cells *in vivo*. A.** HE, Ki-67 and CEA staining of patient tumor tissue (upper) and corresponding patient-derived organoids (bottom). **B.** Green fluorescence expression in cells following transfection with a GFP vector virus. **C.** Transduction efficiency of different AAV9 virus groups was assessed by Western Blot analysis**. D.** Representative images of colons in different groups (n=5 for each group); wild type, AAV-vector + DSS, AAV-PJA2 + DSS, AAV-vector + DSS + mouse interferon injection, AAV-PJA2 + DSS + mouse interferon injection.

**E and F.** The relative body weight changes **(E)** and the number of colonic tumors smaller than 2 mm and at least 2 mm per colon **(F)** of indicated groups. **G.** Representative images of colons in different groups (n=5 for each group); wild type, AAV-sh-NC + DSS, AAV-sh-PJA2#1 + DSS, AAV- sh-NC + DSS + mouse interferon injection, AAV- sh-PJA2#1 + DSS + mouse interferon injection. **H and I.** The relative body weight changes **(H)** and the number of colonic tumors smaller than 2 mm and at least 2 mm per colon **(I)** of indicated groups. Data are shown as mean ± SD of three independent experiments, *P<0.05, **P<0.01, ***P<0.001, ****P<0.0001.

**Figure S4. The expression and enrichment analysis of PJA2 in public databases.** **A, C and E.** The expression of PJA2 was positively correlated with the expression of the IFIT family in colon (left) and rectum adenocarcinoma (right). The expression of PJA2 and the IFIT family was measured by the Chipbase database. **B, D and F.** The expression of HDAC2 was negatively correlated with the expression of the IFIT family in colon (left) and rectum adenocarcinoma (right). The expression of HDAC2 and the IFIT family was measured by the Chipbase database. **G and H.** Gene set enrichment analysis (GSEA) of the TCGA COAD and READ cohort stratified by the mean cut-off value of PJA2 expression. All data are presented as mean±SD. **P<0.01, ***P<0.001, ****P<0.0001.

**Figure S5. PJA2 enhances the tumor suppression function of interferon *in vitro*. A and B.** CCK8 assays were applied to evaluate the viability of PJA2 knockdown **(A)** or overexpression **(B)** cells with the treatment of interferon. **C-D.** Colony formation assays were used to detect proliferation ability of PJA2 knockdown(**C**) and PJA2 overexpressed (**D**) cells with specific treatment. **E-F.** Cells with specific treatment were treated with serum-free medium for 36 hours, and Flow cytometry was used to probe the apoptotic rates (LR+UR) of PJA2 knockdown cells (**E**) and PJA2 overexpression cells (**F**). All data are shown as the means ± SD of three independent experiments and a P-value under 0.05 was considered to be statistically significant. nsP > 0.05, **P < 0.01, ***P < 0.001, ****P<0.0001.

**Figure S6. PJA2 enhances the tumor suppression function of interferon *in vivo*. A.** Representative images of subcutaneous xenograft tumors (n=5 for each group) were obtained from nude mice after the overexpression of PJA2 and injection of interferon. **B.** The analysis of tumor weights of indicated groups. **C.** The tumor volumes were measured every five days. **D.** Representative photographs of H&E, IHC and Tunel staining in xenograft tumors. The protein levels of Ki67 and PJA2 in xenograft tumors were detected by IHC. **E.** The body weight analysis of colons (left) and the Kaplan–Meier survival analysis (right) in indicated groups. **F.** The body weight analysis of colons (left) and the Kaplan–Meier survival analysis (right) in indicated groups. All data are shown as the means ± SD of three independent experiments and a P-value under 0.05 was considered to be statistically significant. ns P > 0.05, **P < 0.01, ***P < 0.001, ****P<0.0001.

**Figure S7. The physical interaction between PJA2 and HDAC2. A.** List of the top 10 differentially expressed proteins identified by mass spectrometry. **B.** HDAC2 was identified by mass spectrometry analysis. **C-D.** SW480 and DLD-1 cells were treated with MG132 (10 μM) for 8 h and then harvested. Cell lysates were analyzed by co-IP followed by western blotting. **E.** IP and WB analyses showing the interactions between FLAG-tagged truncated PJA2 and Myc-tagged HDAC2 proteins in HEK293T cells. Cell extracts were IP with an anti-Myc Ab. **F.** IP and WB analyses showing the interactions between FLAG-tagged truncated PJA2 and Myc-tagged HDAC2 proteins in HEK293T cells. Cell extracts were IP with an anti-Flag Ab. **G.** Detailed hydrogen bonding sites between amino acids in PJA2 and HDAC2. **H.** Docked positions of PJA2 and HDAC2 and design of the mutations of binding sites between PJA2 and HDAC2. All data are presented as mean±SD. **P<0.01, ***P<0.001, ****P<0.0001.

**Figure S8. PJA2 promotes HDAC2 ubiquitination and degradation. A-B.** The relative mRNA expression of HDAC2 after the manipulation of PJA2 in four cells. **C-D.** HDAC2 expression levels in HCT116 and RKO cells treated with cycloheximide (CHX) for the indicated times (top) and relative HDAC2 protein levels (bottom). **E.** HCT116 cells were transfected with plasmids encoding Myc-tagged HDAC2 and the indicated amounts of Flag-tagged PJA2 for 24 h. Cell lysates were analyzed by western blot with indicated antibodies. **F.** Ubiquitination of HDAC2 in SW480 (left), RKO (middle) and HCT116 (right) cells treated with or without MG132 after the manipulation of PJA2. All data are presented as mean±SD. **P<0.01, ***P<0.001, ****P<0.0001.

**Figure S9. The tumor suppression of PJA2 depends on HDAC2. A-B.** In SW480 cells with HDAC2 knockdown, the mRNA expression levels the IFIT family were detected by qPCR after PJA2 knockdown (**A**) and PJA2 overexpression (**B**). **C.** The protein expression of IFIT2 was detected by Western blotting following the transfection of wild-type or mutant PJA2. **D.** CCK8 assays were applied to evaluate the viability of SW480 and RKO cells after the corresponding treatment. **E.** Colony formation assays were used to detect the cell proliferation ability of SW480 and RKO cells after the corresponding treatment. **F.** EdU staining assays were conducted to assess the proliferation ability of SW-480 and RKO cells after the corresponding treatment. **G.** Flow cytometry was used to probe the apoptotic rates (LR+UR) of RKO cells with specific treatment. All data are presented as mean±SD. **P<0.01, ***P<0.001, ****P<0.0001.

**Figure S10. The overexpression of HDAC2 can restore the tumor suppression effect of PJA2 in CRC. A.** CCK8 assays were applied to evaluate the viability of HCT-116 (upper) and DLD-1 cells (bottom) after the corresponding treatment. **B.** Colony formation assays were used to detect the cell proliferation ability of HCT-116 and DLD-1 cells after the corresponding treatment. **C.** EdU staining assays were conducted to assess the proliferation ability of HCT-116 (upper) and DLD-1 cells (bottom) after the corresponding treatment. **D.** Flow cytometry was used to probe the apoptotic rates (LR+UR) of HCT-116 cells with specific treatment. All data are presented as mean±SD. **P<0.01, ***P<0.001, ****P<0.0001.

**Figure S11. The clinical relevance of the PJA2/HDAC2 axis in CRC. A.** The correlational analysis between PJA2 and HDAC2 IHC score in cohort TMA. **B.** The correlational analysis between PJA2 and IFIT2 IHC score in cohort TMA. **C.** The overview of PJA2 expression in cohort TMA. **D.** The overview of HDAC2 expression in cohort TMA. **E.** The overview of IFIT2 expression in cohort TMA.
